# Synthetic Light-Activated Ion Channels for Optogenetic Activation and Inhibition

**DOI:** 10.3389/fnins.2018.00643

**Published:** 2018-10-02

**Authors:** Sebastian Beck, Jing Yu-Strzelczyk, Dennis Pauls, Oana M. Constantin, Christine E. Gee, Nadine Ehmann, Robert J. Kittel, Georg Nagel, Shiqiang Gao

**Affiliations:** ^1^Julius-von-Sachs-Institute, University of Würzburg, Würzburg, Germany; ^2^Neurobiology and Genetics, Theodor-Boveri Institute, Biocenter, University of Würzburg, Würzburg, Germany; ^3^Institute for Synaptic Physiology, University Medical Center Hamburg-Eppendorf, Hamburg, Germany; ^4^Department of Neurophysiology, Institute of Physiology, University of Würzburg, Würzburg, Germany; ^5^Department of Animal Physiology, Institute of Biology, Leipzig University, Leipzig, Germany; ^6^Carl-Ludwig-Institute for Physiology, Leipzig University, Leipzig, Germany

**Keywords:** optogenetics, calcium, potassium, cAMP, bPAC, CNG channel, *Drosophila melanogaster* motoneuron, rat hippocampal neurons

## Abstract

Optogenetic manipulation of cells or living organisms became widely used in neuroscience following the introduction of the light-gated ion channel channelrhodopsin-2 (ChR2). ChR2 is a non-selective cation channel, ideally suited to depolarize and evoke action potentials in neurons. However, its calcium (Ca^2+^) permeability and single channel conductance are low and for some applications longer-lasting increases in intracellular Ca^2+^ might be desirable. Moreover, there is need for an efficient light-gated potassium (K^+^) channel that can rapidly inhibit spiking in targeted neurons. Considering the importance of Ca^2+^ and K^+^ in cell physiology, light-activated Ca^2+^-permeant and K^+^-specific channels would be welcome additions to the optogenetic toolbox. Here we describe the engineering of novel light-gated Ca^2+^-permeant and K^+^-specific channels by fusing a bacterial photoactivated adenylyl cyclase to cyclic nucleotide-gated channels with high permeability for Ca^2+^ or for K^+^, respectively. Optimized fusion constructs showed strong light-gated conductance in *Xenopus laevis* oocytes and in rat hippocampal neurons. These constructs could also be used to control the motility of *Drosophila melanogaster* larvae, when expressed in motoneurons. Illumination led to body contraction when motoneurons expressed the light-sensitive Ca^2+^-permeant channel, and to body extension when expressing the light-sensitive K^+^ channel, both effectively and reversibly paralyzing the larvae. Further optimization of these constructs will be required for application in adult flies since both constructs led to eclosion failure when expressed in motoneurons.

## Introduction

With the discovery of channelrhodopsin-1 (Nagel et al., 2002) and the demonstration of light-induced membrane depolarization via ChR2 (Nagel et al., 2003), optical manipulation of cell physiology with transgenic photoreceptors became the method of choice for manipulating genetically defined cells (Boyden et al., 2005; Li et al., 2005; Nagel et al., 2005; Bi et al., 2006); (Ishizuka et al., 2006). The opsin-based toolbox has expanded and includes the earlier discovered and characterized pump rhodopsins (Zhang et al., 2007; Chow et al., 2010), engineered channel rhodopsins (Kleinlogel et al., 2011; Lin et al., 2013; Dawydow et al., 2014; Scholz et al., 2017), and more recently, light-gated anion channels and nucleotidyl cyclases (Klapoetke et al., 2014; Gao et al., 2015; Govorunova et al., 2015; Scheib et al., 2015).

The optogenetic toolkit is not restricted to rhodopsins. The first optogenetic application employing a light-activated enzyme was light-induced increase of cytosolic cAMP with the photoactivated adenylyl cyclases PACα and PACβ (Schröder-Lang et al., 2007). These flavoproteins with a BLUF domain (blue light using FAD) were discovered in the unicellular flagellate *Euglena gracilis* (Iseki et al., 2002). Several years later, in the genome of the soil bacterium *Beggiatoa*, a smaller photoactivated adenylyl cyclase (bPAC) was found and characterized (Ryu et al., 2010; Stierl et al., 2011).

Despite the success of ChR2 and certain mutants, there are also some limitations. ChR2 is a non-selective cation channel and its Ca^2+^ permeability and single channel conductance are low (Nagel et al., 2003; Kleinlogel et al., 2011). The point mutation L132C enhanced its Ca^2+^ permeability which is, however, still weaker than for H^+^, Na^+^, and K^+^ (Kleinlogel et al., 2011). Therefore, a more conductive light-sensitive channel with high Ca^2+^ permeability is of interest. Airan et al. generated chimeric OptoXRs from rhodopsin and GPCRs (G-protein coupled receptors) to manipulate intracellular second messengers and further regulate downstream ion channel activity (Airan et al., 2009). Recently introduced optogenetic tools for Ca^2+^ manipulation such as OptoSTIM1 (Kyung et al., 2015) or Opto-CRAC (He et al., 2015) are based on the interaction of light-regulated STIM1 and CRAC (Ca^2+^ release-activated Ca^2+^) channel. This method requires endogenous CRAC to be regulated by engineered STIM1. These tools are slow and highly dependent on background levels of STIM and CRAC.

As inhibitory tools, highly efficient Cl^−^ conducting anion channelrhodopsins (ACRs) have been introduced (Govorunova et al., 2015), but whether they hyperpolarize or depolarize cells depends on the intracellular Cl^−^ concentration (Mahn et al., 2016; Wiegert and Oertner, 2016). A light-gated K^+^ channel is therefore highly desirable for light-induced hyperpolarization. Already in 2004, Banghart et al. designed a light activated K^+^ channel with addition of a photoisomerizable azobenzene (Banghart et al., 2004). The light-sensitive light-oxygen-voltage (LOV) domain had also been applied to control the K^+^ channel together with a peptide toxin (Schmidt et al., 2014). Also to this end, the light-gated potassium channel BLINK1 was designed, by fusing the photosensory domain LOV2-Jα from the oat photoreceptor phototropin with the small viral K^+^ channel Kcv (Cosentino et al., 2015). BLINK1 has advantages as it is small and requires only the ubiquitous chromophore flavin mononucleotide (FMN). BLINK1 has been expressed in HEK293 cells and zebrafish. However, in our hands the photocurrent of BLINK1, expressed in *Xenopus* oocytes is almost undetectable. This might be due to low expression or poor plasma membrane targeting (data not shown).

In this study, we generated light-gated Ca^2+^ permeant and K^+^ selective channels by fusing bPAC to cyclic nucleotide-gated (CNG) channels. Light-gated cAMP production from bPAC leads to activation of the CNG channel. The bovine olfactory organ CNG channel mutant T537S (OLF for short) is highly Ca^2+^ permeant (Altenhofen et al., 1991; Frings et al., 1995; Dzeja et al., 1999) and SthK from *Spirochaeta thermophila* (Brams et al., 2014; Kesters et al., 2015) is a selective K^+^ channel with a single channel conductance of 71 picosiemens (Brams et al., 2014).

Combining OLF and SthK with bPAC showed strong light-gated conductance in *Xenopus* oocytes. Fusing bPAC to CNG as one construct facilitated the subsequent transgenic handling, showed faster kinetics and required less cAMP for channel opening than the co-expressed proteins, presumably because of the close spatial proximity of cyclase and channel. These constructs were also effective in hippocampal neurons depolarizing or blocking spiking, respectively. Expression in *Drosophila* motoneurons allowed us to light-control the motility of larvae. Illumination led to body contraction with the OLF fusion construct, and to body extension with the SthK fusion construct. Thus, we have engineered new optogenetic tools that depolarize and increase intracellular Ca^2+^ or hyperpolarize cells and demonstrate that they can be used to activate and inhibit neurons, respectively.

## Materials and methods

### Molecular biology

The bovine olfactory organ CNG (*Bos taurus* CNGA2) channel mutant T537S was already used in a previous study with PACα and PACβ from *E. gracilis* (Schröder-Lang et al., 2007). The bPAC sequence is as previously published (Stierl et al., 2011). The SthK channel DNA sequence was synthesized by GeneArt Strings DNA Fragments (Life technologies, Thermo Fisher Scientific) according to the published amino acid sequence (Brams et al., 2014; Kesters et al., 2015) with codon usage optimized for *Mus musculus*. The DNA fragments were ligated and inserted into the oocyte expression vector pGEM-HE within N-terminal BamHI and C-terminal HindIII restriction sites. For the fly transgenic vector, the DNA insert was ligated into the KpnI and BamHI restriction sites of the expression vector pJFRC7, instead of ChR2-XXL (Dawydow et al., 2014).

Sequences were confirmed by complete DNA sequencing (GATC Biotech). Exact DNA sequences of all different constructs are shown in the Supplementary Data Sheet 1. Plasmids were linearized by NheI digestion. cRNAs were generated by *in vitro* transcription with the AmpliCap-MaxT7 High Yield Message Maker Kit (Epicentre Biotechnologies), using the linearized plasmid DNA as template.

### *Xenopus* oocyte expression and two electrode voltage clamp recording

*Xenopus* oocytes were injected with *in-vitro* generated cRNA and maintained at 16°C in ND96 solution: 96 mM NaCl, 2 mM KCl, 1 mM CaCl_2_, 1 mM MgCl_2_, 10 mM HEPES, pH 7.4, and 50 μg/mL gentamycin. Injected oocytes were incubated at 16°C for 3 days.

Electrophysiological measurements with *Xenopus* oocytes were performed in Standard Ringer's solution (110 mM NaCl, 5 mM KCl, 2 mM BaCl_2_, 1 mM MgCl_2_, 10 mM HEPES, pH 7.4), unless specified. A 532 nm laser and a 473 nm laser (Changchun New Industries Optoelectronics Tech) were used as light sources. The light intensities at different wavelengths were measured with a Laser Check optical power meter (Coherent Inc.). Currents were measured at room temperature (20–23°C) with a two-electrode voltage clamp amplifier (TURBO TEC-03X, npi electronic GmbH, Tamm, Germany). Electrode capillaries (Φ = 1.5 mm, wall thickness 0.178 mm, Hilgenberg) were filled with 3 M KCl, with tip openings yielding a resistance of 0.4–1 MΩ. Stimulation and data acquisition were controlled with an AD-DA converter (Digidata 1322A, Axon Instruments) and WinWCP software (v4.1.7, Strathclyde University, United Kingdom).

### Rat hippocampal neuron expression, electrophysiology, and imaging

Single-cell electroporation was used to introduce plasmid DNA into rat hippocampal neurons in organotypic slice cultures prepared from P5-P7 Wistar rats (Janvier), as described (Gee et al., 2017; Wiegert et al., 2017). Neurons were electroporated with plasmids encoding either OLF-T-YFP-bPAC-Ex (50 ng/μl) or SthK-T-YFP-bPAC-Ex (100 ng/μl), together with mKate2 (10 ng/μl).

After allowing 3 (SthK-T-YFP-bPAC-Ex) to 6 (OLF-T-YFP-bPAC-Ex) days for expression, slices were transferred to the perfusion chamber of an upright microscope (Olympus BX61WI) fitted with an LED (Mightex Systems), which was coupled through the camera port using a multimode fiber (1.0 mm) and collimator (Thorlabs) to photostimulate through the 40 × water immersion objective (Plan-Apochromat, 40 × 1.0 numerical aperture, Zeiss). Radiant power was determined using a silicon photodiode (Newport) positioned in the specimen plane and divided by the illuminated field (0.244 mm^2^).

The extracellular solution contained (in mM): NaCl 119, NaHCO_3_ 26.2, D-glucose 11, KCl 2.5, NaH_2_PO_4_ 1, MgCl_2_ 4, CaCl_2_ 4, pH 7.4, 310 mOsm/kg, saturated with 95% O_2_/5% CO_2_. Recording temperature was 28–30°C. The following were added to the perfusate to block synaptic activity unless otherwise indicated: NBQX 10 μM, CPPene 10 μM, picrotoxin 100 μM (Tocris). Wash-in of the antagonists did not affect the light evoked currents. The intracellular solution contained (in mM): K-gluconate 135, HEPES 10, EGTA 0.2, Na_2_-ATP 4, Na-GTP 0.4, Magnesium chloride (MgCl_2_) 4, ascorbate 3, Na_2_-phosphocreatine 10, pH 7.2, 295 mOsm/kg. The liquid junction potential was measured (−14.4 mV) and compensated. Patch electrodes were made from thick-walled borosillicate glass and had resistances of 3–5 MΩ. Neurons were voltage-clamped at −70 mV using an Axopatch 200B amplifier. National Instruments A/D boards and Ephus software were used to record and control the experiment. Series resistance was <15 MΩ and was not compensated during voltage clamp recordings. The bridge balance compensation circuitry was used during current clamp recordings.

### *Drosophila* culture conditions and stocks

The following strains were generated in this study:

RJK 342, y[1] w[1118]; [UAS-OLF-T-YFP-bPAC-Ex]/CyO; +
LAT388, y[1] w[1118]; [UAS-SthK-T-YFP-bPAC-Ex]/CyO; +
RJK560, y[1] w[1118]; [UAS-CD8-YFP-bPAC]/CyO; +
RJK 564, y[1] w[1118]; [UAS-OLF-T-YFP-Ex]/CyO; +

Transgenic flies carrying UAS-OLF-T-YFP-bPAC-Ex, UAS-SthK-T-YFP-bPAC-Ex, UAS-OLF-T-YFP-Ex and UAS-CD8-YFP-bPAC were generated by targeted PhiC31 recombinase-mediated insertion into the genomic P[acman] landing site attP-9A[VK18] located on the second chromosome (Venken et al., 2006) (BestGene Inc.).

All larvae were reared on standard cornmeal/agar medium at 25°C and 70% relative humidity in constant darkness.

Abbreviations used in the figures for larvae of different genotypes were as below:

OLF-bP = UAS-**OLF**-T-YFP-**bP**AC-Ex/ok6-Gal4;
SthK-bP = UAS-**SthK**-T-YFP-**bP**AC-Ex/ok6-Gal4;
OLF = UAS-**OLF**-T-YFP-Ex/ok6-Gal4;
CD8-bP = ok6-Gal4/UAS-**CD8**-YFP-**bP**AC;
Ctrl-G = +/ok6-**G**al4;
Ctrl-O = UAS-**O**LF-T-YFP-bPAC-Ex/+;
Ctrl-S = UAS-**S**thK-T-YFP-bPAC-Ex/+.

### Recording larval locomotor activity

For recording larval locomotion, 5–6 day old third instar larvae were selected and placed on a circular disc of 1.5% agarose, 85 mm in diameter. The agarose disk was seated on top of a FIM recording setup, built as previously described (Risse et al., 2013). Larval locomotion was recorded by an infrared-camera underneath, picking up the infrared light waves scattered by the larva and the agarose disk. The camera resolution was 2,592 × 1,944 pixels, the recording frame-rate was set to one frame per second.

Blue light illumination was applied through an LED array (470 nm, light intensities applied are indicated in figure legends). Control conditions were recorded under ambient red room lighting (620 nm, 0.1 μW/cm^2^). LED spectrum was determined using the QE65000 Spectrometer (Ocean Optics, Dunedin FL 34698) and intensity was measured using the Laser Check optical power meter (Coherent Inc.).

The open source tracking software FIMtrack (Risse et al., 2017) was used for tracking and analyzing various parameters of larval locomotion. We used two parameters “body length” (distance from larval head to tail) and “momentum distance” (distance between the larval center of mass from one recording frame to the next). The average momentum distance divided by the recording frame rate yields the velocity.

### cAMP assay with *Xenopus* oocytes and *Drosophila* larvae

*Xenopus* oocytes injected with different constructs were incubated at 16°C for 3 days in ND96. Oocytes were either kept in the dark or illuminated for 20 s with blue light (473 nm, 0.3 mW/mm^2^). 4–6 oocytes injected with the same construct were homogenized by simply pipetting with a 20–200 μl pipette in Sample Diluent (containing 0.1 N HCl and pH indicator, Arbor Assays). Samples were then centrifuged at 10,000 rpm for 10 min at room temperature. The supernatant was collected for cAMP assays.

*Drosophila* larvae were prepared in the dark or after 20 min blue light illumination (473 nm, 0.3 mW/mm^2^). Larvae in the dark or after illumination were frozen immediately in liquid N_2_. The larvae were then homogenized with the micropestle for eppitubes (Eppendorf). Sample Diluent was used to suspend the ground samples. Samples were heated up to 95°C for 5 min and then centrifuged 10 min at 10,000 rpm room temperature for cAMP assays.

cAMP concentrations in the prepared samples were determined using DetectX High Sensitivity Direct Cyclic AMP Chemiluminescent Immunoassay Kit (Arbor assays).

### Imaging

Fluorescence images of *Xenopus* oocytes were taken 3 days after injection with a confocal microscope (Leica DM6000). Movies of *Drosophila* larvae were obtained with a Leica DMi8 fluorescence microscope. Images of pupae and flies were obtained with a Keyence digital microscope equipped with VH-Z20 (20-200X) Ultra Small High-performance Zoom Lens.

Images of transfected hippocampal neurons were acquired with a confocal laser scanning microscope (Olympus FLUOVIEW FV1000). The signal from YFP and mKate2 were acquired individually by using the 488 nm (YFP) and 559 nm (mKate2) laser lines, emission channels used were 495–595 nm (YFP) and 565–665 nm (mKate2).

### Immunohistochemistry

Staining of *Drosophila* larval NMJs and VNCs followed a standard protocol (Ljaschenko et al., 2013). In brief, male third instar larvae were dissected in Ca^2+^-free HL-3 (Haemolymph-like solution) (Stewart et al., 1994), fixed in 4% paraformaldehyde for 10 min at room temperature and blocked with PBT (0.05% Triton X-100; T8787, Sigma Aldrich) supplemented with 5% normal goat serum (G9023, Sigma Aldrich) for 30 min. The primary antibody, rabbit anti-GFP (1:400; A11122, Invitrogen), was diluted in blocking solution and incubated at 4°C overnight. This was followed by 2 short and 3 × 20 min washing steps with PBT and incubation with secondary antibodies, goat anti-rabbit-AlexaFluor488 (1:250; A11034, Invitrogen) and HRP-Cy3 (1:250; 123-165-021, Dianova) in blocking solution for 2 h at room temperature. Subsequently, samples were washed (2 short, 3 × 20 min washing steps) before mounting in Vectashield (H-1000, Vector laboratories). Images were acquired with a Zeiss Imager.Z2 confocal system (objective: 63x, numerical aperture 1.4, oil). For visualization, HRP-signals of NMJs were background subtracted, blurred (Gauss blur σ = 1 px) and normalized (ImageJ, National Institutes of Health). For VNCs, only a subset of the whole confocal stack was maximum projected. All genotypes were stained in the same vial.

### Data analysis

OriginPro 2017 (OriginLab Corporation, Northampton, MA, United States) and Microsoft Excel were used for oocyte and *Drosophila* data analysis. All data are expressed as mean ± standard error of the mean (SEM) or standard deviation (SD), as indicated. Paired Student's *t*-tests were used for statistical comparisons for all data other than larval behavior. For larval locomotion data, *p*-values of datasets were calculated using R. First each dataset was tested for normal gaussian distribution using a Shapiro-Wilk test. If all data of one dataset followed normal gaussian distribution, a pairwise-*t*-test was performed with Bonferroni correction. If at least one of the tested datasets did not follow normal gaussian distribution, a pairwise Wilcox-test was performed with Bonferroni correction.

Differences were considered significant ^***^p < 0.001, ^**^p < 0.01, ^*^p < 0.05.

Data from hippocampal neurons were analyzed with custom routines programmed in MATLAB. Graphs, curve-fitting and non-parametric statistical analyses of the neuron data were performed with GraphPad Prism 6.0. Data are shown as median and interquartile range.

### Ethics statement

The laparotomy to obtain oocytes from *Xenopus laevis* was carried out in accordance with the principles of the Basel Declaration and recommendations of Landratsamt Wüerzburg Veterinaeramt. The protocol under License #70/14 from Landratsamt Wüerzburg, Veterinaeramt, was approved by the responsible veterinarian. Rats were housed and bred at the University Medical Center Hamburg animal facility. All procedures were performed according to protocols approved by the Behörde für Gesundheit und Verbraucherschutz (BGV), Hamburg.

## Results

### Generation and optimization of a light-gated highly Ca^2+^-permeant cation channel

**Figure 1:**
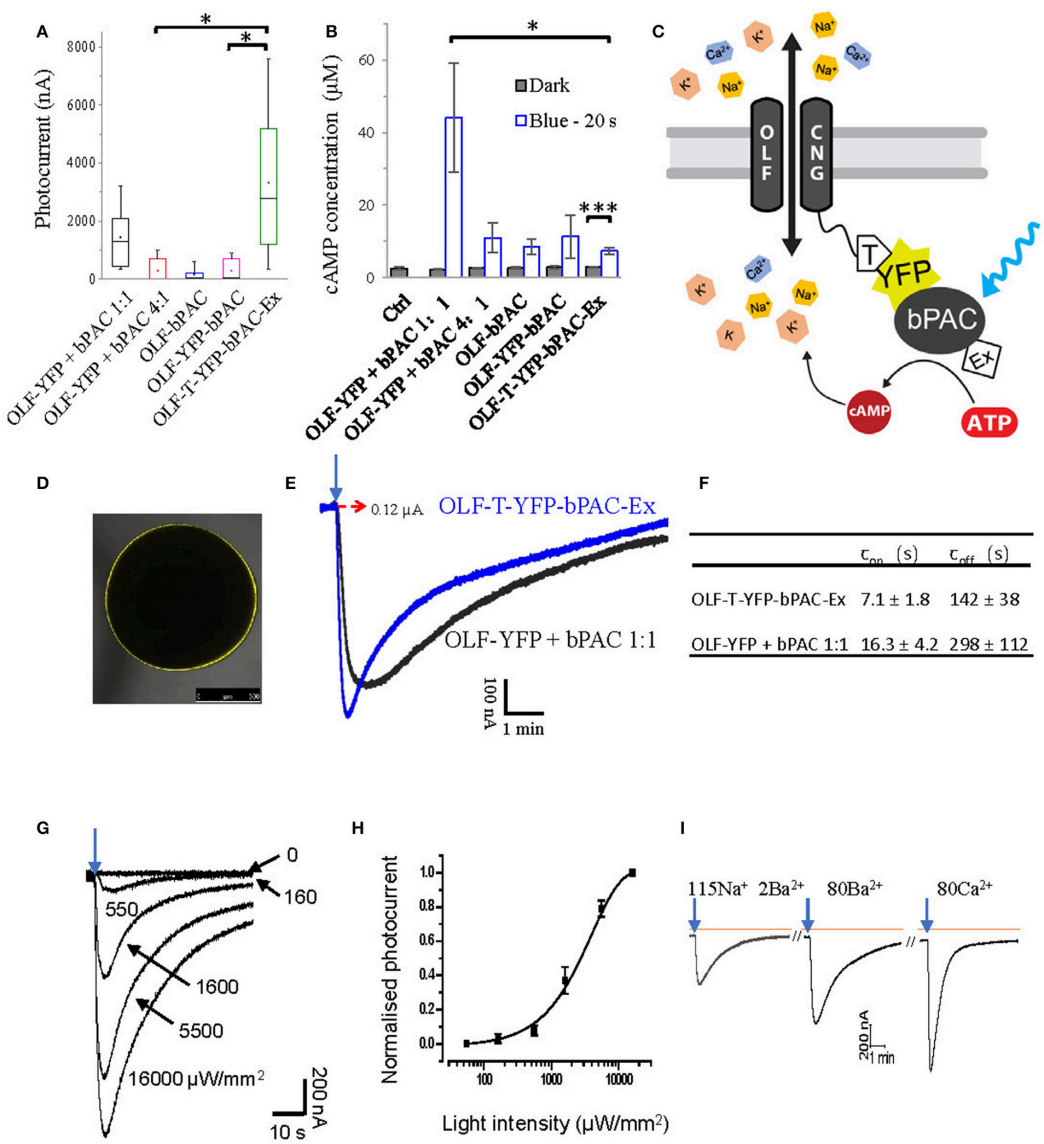
Design of a light-gated Ca^2+^-permeant cation channel. **(A)** Photocurrents of *Xenopus* oocytes expressing the bovine olfactory cyclic nucleotide gated channel (OLF) and the photoactivatable adenylyl cyclase (bPAC) and various fusion constructs. Total cRNA amounts were adjusted to keep the copies of injected OLF constant. Ratios of cRNA mixtures are indicated. Experiments were performed employing blue light illumination (0.2 s, 1 mW/mm^2^, 473 nm, *n* = 6). **(B)** cAMP production of different OLF and bPAC fusion constructs or mixes; blue light (473 nm, 0.3 mW/mm^2^); *n* = 3 experiments, each with 6 oocytes; error bars = SD. **(C)** Schematic of the designed OLF (T537S) and bPAC fusion construct. T, plasma membrane trafficking signal; Ex, ER export signal. **(D)** Fluorescence picture of *Xenopus* oocyte expressing OLF-T-YFP-bPAC-Ex. **(E)** Example photocurrent of OLF-T-YFP-bPAC-Ex and 1:1 mixture of OLF and bPAC. Holding potential −60 mV; illumination with 473 nm blue light, 1 mW/mm^2^, red dashed arrow indicates 0 nA. **(F)** Kinetics of photocurrents in oocytes expressing OLF-T-YFP-bPAC-Ex and 1:1 mixture of OLF and bPAC. Light intensity was adjusted to evoke currents of ~0.6 μA. *n* = 3, error bars = SD. **(G)** Example OLF-T-YFP-bPAC-Ex photocurrent traces from 1 oocyte induced by 1 s 473 nm light of different intensities, ~15 min recovery time in the dark for each illumination. **(H)** OLF-T-YFP-bPAC-Ex photocurrents induced by light of different intensities. *n* = 4, error bars = SD. **(I)** A current recording trace from an oocyte expressing OLF-T-YFP-bPAC-Ex with 1 s blue light (473 nm, 550 μW/mm^2^) illumination and switching bath solutions containing different cations (115Na 2Ba^2+^: 115 mM NaCl, 2 mM BaCl_2_; 80Ba^2+^: 80 mM BaCl_2_; 80Ca^2+^: 80 mM CaCl_2._ All buffers contain 1 mM MgCl_2_, 5 mM HEPES and pH adjusted to 7.6). Orange line indicates basal current, arrows indicate light pulse, ~15 min recovery time in the dark between each illumination.

Previously, soluble bPAC was shown to activate CNG channels, when co-expressed in *Xenopus* oocytes and hippocampal neurons (Stierl et al., 2011). Co-expression of the CNG channel OLF/T537S from the bovine olfactory organ (abbreviated OLF) together with bPAC in oocytes generated a pronounced inward photocurrent when exposed to a short light flash [473 nm, 1 mW/mm^2^, 0.2 s, Figure 1A, OLF-YFP + bPAC (1:1)]. As the bovine OLF channel highly fluxes Ca^2+^(Frings et al., 1995; Dzeja et al., 1999), recordings were performed with Ba^2+^ instead of Ca^2+^ as the permeant ion to prevent activation of endogenous Ca^2+^-activated Cl^−^ channels in the oocytes.

We then tested several OLF and bPAC fusion constructs in oocytes. Very few oocytes expressing bPAC directly attached to the C-terminus of OLF had photocurrents, therefore the averaged photocurrent is very small (Figure 1A, OLF-bPAC). Inserting a YFP domain between the channel and bPAC did not improve this significantly (Figure 1A, OLF-YFP-bPAC) although light induced cAMP production by both OLF-bPAC and OLF-YFP-bPAC (Figure 1B). We hypothesized that the plasma membrane trafficking of the channel might be hampered when fusing bPAC or YFP-bPAC to the C terminus of OLF.

Therefore, we introduced a plasma membrane trafficking signal (T, AA sequence: KSRITSEGEYIPLDQIDINV) between the OLF channel and YFP and added an ER export signal (Ex, AA sequence: FCYENEV) (Gradinaru et al., 2010) to the C-terminal end, as shown in Figure 1C. The T and Ex sequences were used to improve the plasma membrane targeting of halorhodopsin and bacteriorhodopsin in mammalian neurons effectively (Gradinaru et al., 2010). The new construct, OLF-T-YFP-bPAC-Ex, showed good expression in *Xenopus* oocytes (Figure 1D) and larger photocurrents than the other fusion constructs or the co-expressed OLF channel and soluble bPAC (Figure 1A). Interestingly, when illuminated, OLF-T-YFP-bPAC-Ex also produced the smallest increase in cytosolic cAMP (Figure 1B).

To compare the kinetics of OLF-T-YFP-bPAC-Ex photocurrents with the kinetics of the co-expressed OLF and bPAC (OLF-YFP + bPAC 1:1), photocurrents of ~600 nA were elicited. The fusion construct showed faster onset and offset of the photocurrents than did the co-expression of OLF-YFP and soluble bPAC (Figures 1E,F). The half-saturation light intensity of Olf-T-YFP-bPAC-Ex with 1 s light flash was determined to be ~2.8 mW/mm^2^ (Figures 1G,H).

OLF is a non-selective cation channel with high Ca^2+^ permeability. In oocytes, it showed high conductance with Ba^2+^, which is a cation most equivalent to Ca^2+^ (Figure 1I). The Ca^2+^ buffer also generated a big current but it should be mixed with the endogenous Ca^2+^- activated Cl^−^ channel current. The faster recovery time in Ca^2+^ buffer suggested the high Ca^2+^ concentration blocking effect of the OLF channel (Frings et al., 1995; Dzeja et al., 1999).

**Figure 2:**
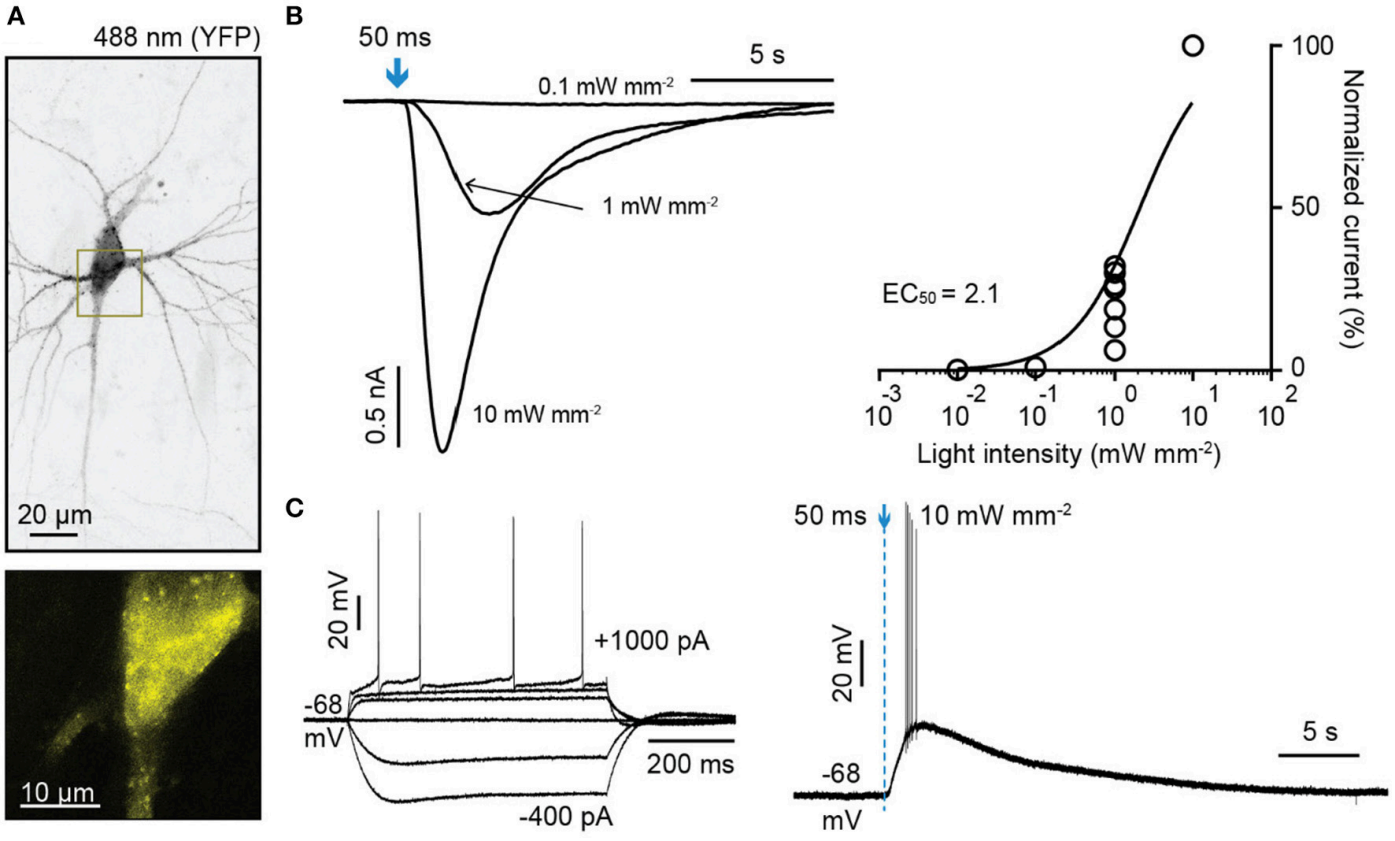
Characterization of light-gated OLF channel in hippocampal neurons. **(A)** Top: Maximum intensity projection of confocal images of hippocampal neurons 6 days after electroporation with DNA encoding OLF-T-YFP-bPAC-Ex (OLF-bP) and mKate2 (excitation 488 nm). Bottom: Single plane of the YFP signal of indicated area in **(A)** (dark yellow box). **(B)** Left: Sample photocurrents evoked by a 50 ms light pulse (470 nm) of different intensity in hippocampal neurons expressing mKate2 and OLF-bP. Right: Light intensity-response relationship fitted with a quadratic equation. Photocurrents are normalized to the maximum current recorded from each neuron. *n* = 6. **(C)** Left: Whole-cell responses to current injections from −400 to 1,000 pA in an OLF-bP expressing hippocampal neuron. Right: Action potentials of the same cell generated by applying a short 470 nm light flash (50 ms at 10 mW/mm^2^).

OLF-T-YFP-bPAC-Ex (OLF-bP) also induced photocurrents in hippocampal pyramidal neurons (Figure 2). The half-saturation light intensity of OLF-bP with a 50 ms light flash in neurons was ~2.1 mW/mm^2^ (Figure 2B). A 50 ms, 10 mW/mm^2^, 470 nm flash slowly depolarized neurons and evoked action potentials (Figure 2C).

### Generation of a light-gated potassium channel

**Figure 3:**
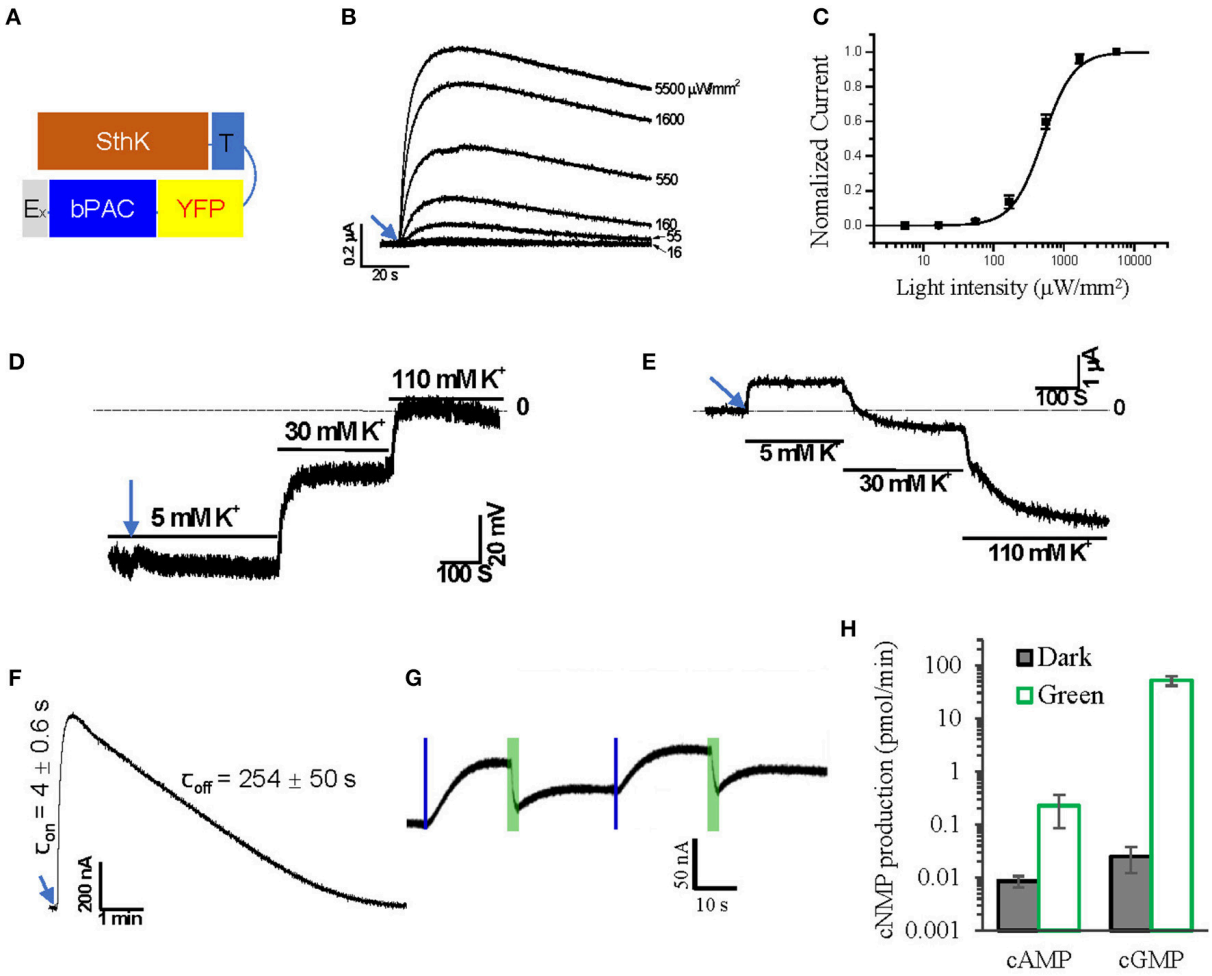
Design of a light-gated potassium channel. **(A)** Schematic of the designed SthK and bPAC fusion construct. **(B)** Representative Photocurrent traces recorded from *Xenopus* oocytes injected with 2 ng SthK-T-YFP-bPAC-Ex (SthK-bP) activated by 1 s blue light (473 nm) of different intensities, ~30 min recovery time in the dark for each illumination. **(C)** Normalized currents against light intensity curve fitted monoexponentially. The half-maximal light intensity value was determined to be 500 μW/mm^2^; *n* = 4, error bars = SEM. **(D)** Representative traces of membrane potential recording while switching bath solutions containing 5, 30, and 110 mM K^+^ after 5 s blue light (473 nm, 550 μW/mm^2^) illumination. **(E)** Representative traces of current recording (holding at −40 mV) while switching bath solutions containing 5, 30 and 110 mM K^+^ after 5 s blue light (473 nm, 550 μW/mm^2^) illumination. **(F)** On and off kinetics of SthK-bP photocurrents in oocytes with 1 s blue light (473 nm, 550 μW/mm^2^) illumination; for values, *n* = 4, error bars = SD. **(G)** Representative photocurrent traces from oocytes co-injected with 1.2 ng SthK-bP and 5 ng BeCyclop cRNA in *Xenopus* oocytes. Currents were induced by 200 ms blue light (473 nm, 550 μW/mm^2^) illumination and reduced by 3 s green light (532 nm, 1 mW/mm^2^) illumination. **(H)** cAMP and cGMP production of *Xenopus* oocyte membranes co-expressing SthK-bP and BeCyclop in the dark or light (532 nm, 80 μW/mm^2^). *n* = 3, error bars = SD.

Recently, a cyclic nucleotide gated K^+^ channel from *S. thermophila*, SthK, was described (Brams et al., 2014; Kesters et al., 2015). We fused SthK with T-YFP-bPAC-Ex to generate a light-gated potassium channel, SthK-T-YFP-bPAC-Ex (Figure 3A, SthK-bP). In 5 mM extracellular K^+^, oocytes expressing SthK-bP had outward photocurrents that increased with increasing light intensities (1 s, 473 nm, 16 to 5,500 μW/mm^2^; Figure 3B). The half-saturation light intensity of SthK-bP with a 1 s light flash was determined to be ~500 μW/mm^2^ (Figure 3C).

We applied 5 s illumination to SthK-bP expressing oocytes, which evoked photocurrents that persisted for several minutes, during which we changed the extracellular K^+^ concentration to verify the K^+^ permeability of the fusion construct. Both membrane voltage (Figure 3D) and the currents recorded at −40 mV (Figure 3E) showed a high dependence on extracellular K^+^, confirming that SthK-bP is highly permeable to K^+^. The opening of SthK-bP is faster than OLF-bP and the closing is slower (Figure 3F). This is in good accordance with the lower EC_50_ of SthK for cAMP (3.7 ± 0.55 μM) (Altenhofen et al., 1991) than that of OLF/T537S (14 ± 4 μM) (Brams et al., 2014).

The SthK channel is reported to be activated by cAMP, with cGMP as an antagonist (Kesters et al., 2015). We therefore co-expressed SthK-bP with *Be*Cyclop, a green light-activated guanylyl cyclase (Gao et al., 2015). A short blue light flash (0.2 s, 550 μW/mm^2^, 473 nm) activated the bPAC and initiated outward currents. Applying green light (3 s, 1 mW/mm^2^, 532 nm, selective for *Be*Cyclop), to increase cGMP, attenuated the currents (Figure 3G). The illumination time and light intensities were adjusted to produce enough cGMP upon green light illumination. However, the cAMP-induced current was not fully blocked and we found that the 532 nm illumination still activated bPAC slightly (Figure 3H).

**Figure 4:**
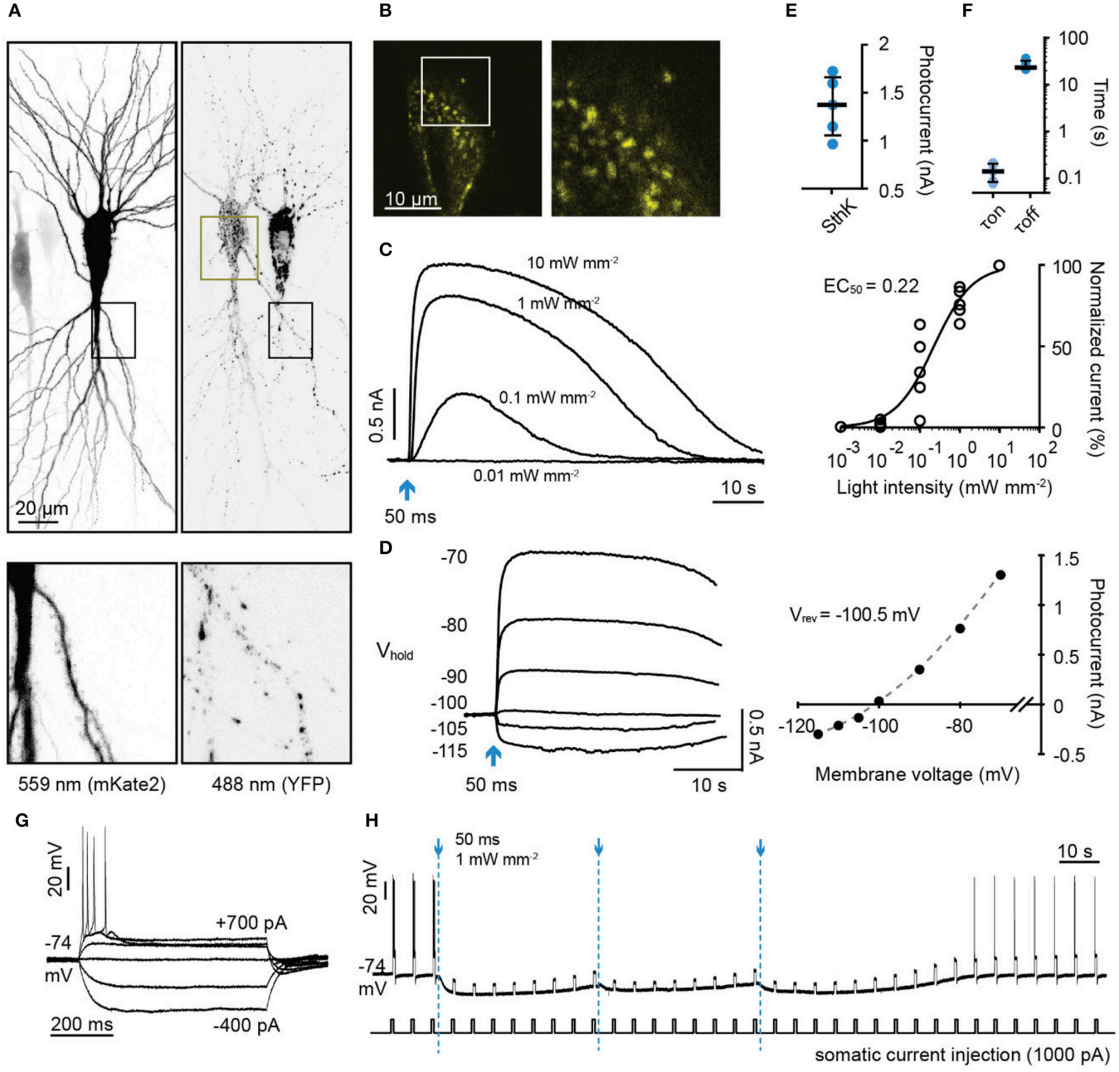
Characterization of a light-gated potassium channel in hippocampal neurons. **(A)** Maximum intensity projection of confocal images of hippocampal neurons 3 days after electroporation with DNA encoding SthK-bP and mKate2 (left: excitation 559 nm; right: excitation 488 nm). Lower images are close up of the regions indicated by dark boxes. **(B)** Single plane of the YFP signal of the region indicated in **(A)** (dark yellow box). **(C)** Left: Sample photocurrents evoked by a 50 ms light pulse (470 nm) of different intensity in a hippocampal neuron expressing mKate2 and SthK-bP. The holding potential was −70 mV. Right: Light intensity-response relationship fitted with a quadratic equation. Photocurrents are normalized to the maximum current recorded in each neuron. *n* = 5. **(D)** Left: Sample traces of photocurrents recorded from a SthK-bP expressing neuron when stimulated with 0.1 mW/mm^2^, 470 nm light (50 ms) while holding the cell at membrane potentials from −70 mV down to −115 mV. Baselines are aligned. Right: Current-voltage plot. A non-linear fit was applied to determine the K^+^ equilibrium potential (−100.5 mV). **(E)** Photocurrent amplitude recorded from neurons expressing mKate2 and SthK-bP when stimulated with 1 mW mm^−2^ 470 nm light. Shown are individual data points, median and 25–75% interquartile range, *n* = 5; median peak current SthK-bP 1.37 nA. **(F)** Kinetics of SthK-bP; data obtained from traces when the stimulation intensity was 1 mW mm^−2^ (470 nm, 50 ms); *n* = 5. **(G)** Whole-cell responses to current injections from −400 to 700 pA in SthK-bP expressing hippocampal neuron. **(H)** Action potentials generated by repeated somatic current injection (1,000 pA, 600 ms, ISI 5 s) were blocked for 3 min by 3 470 nm light flashes at 40 second intervals (50 ms at 1 mW mm^−2^); same neuron as in **(G)**.

We then expressed SthK-bP in rat hippocampal neurons together with the red fluorescent protein mKate2 (Figures 4A,B). Short flashes of blue light (50 ms, 470 nm) induced outward currents that were light intensity-dependent (Figures 4C,E,F). The currents reversed at −100.5 mV close to the calculated reversal potential of −104 for K^+^ (Figure 4D). We then tested whether the hyperpolarization induced by activating SthK-bP would be sufficient to block action potentials (Figure 4G). A single light flash (50 ms, 470 nm, 1 mW/mm^2^) completely blocked action potentials induced by 600 ms 1,000 pA current injections for around 1 min. This complete blockade of action potentials was extended to 3 min by repeatedly flashing blue light at 40 second intervals (Figure 4H). SthK-bP could be activated strongly by 400 and 470 nm light and very slightly by 530 nm light (Supplementary Figure 1).

### Expression of light-gated CNG channels in *Drosophila* larvae

The two optimized constructs for light-induced Na^+^/Ca^2+^ permeability and K^+^-selective permeability, OLF-bP and SthK-bP, were used to generate transgenic flies. The target genes were placed under the control of the UAS promoter to allow crossing with Gal4 lines.

We obtained expression in motoneurons by crossing the UAS-OLF-T-YFP-bPAC-Ex and UAS-SthK-T-YFP-bPAC-Ex flies with the ok6-Gal4 line (Sanyal, 2009).

**Figure 5:**
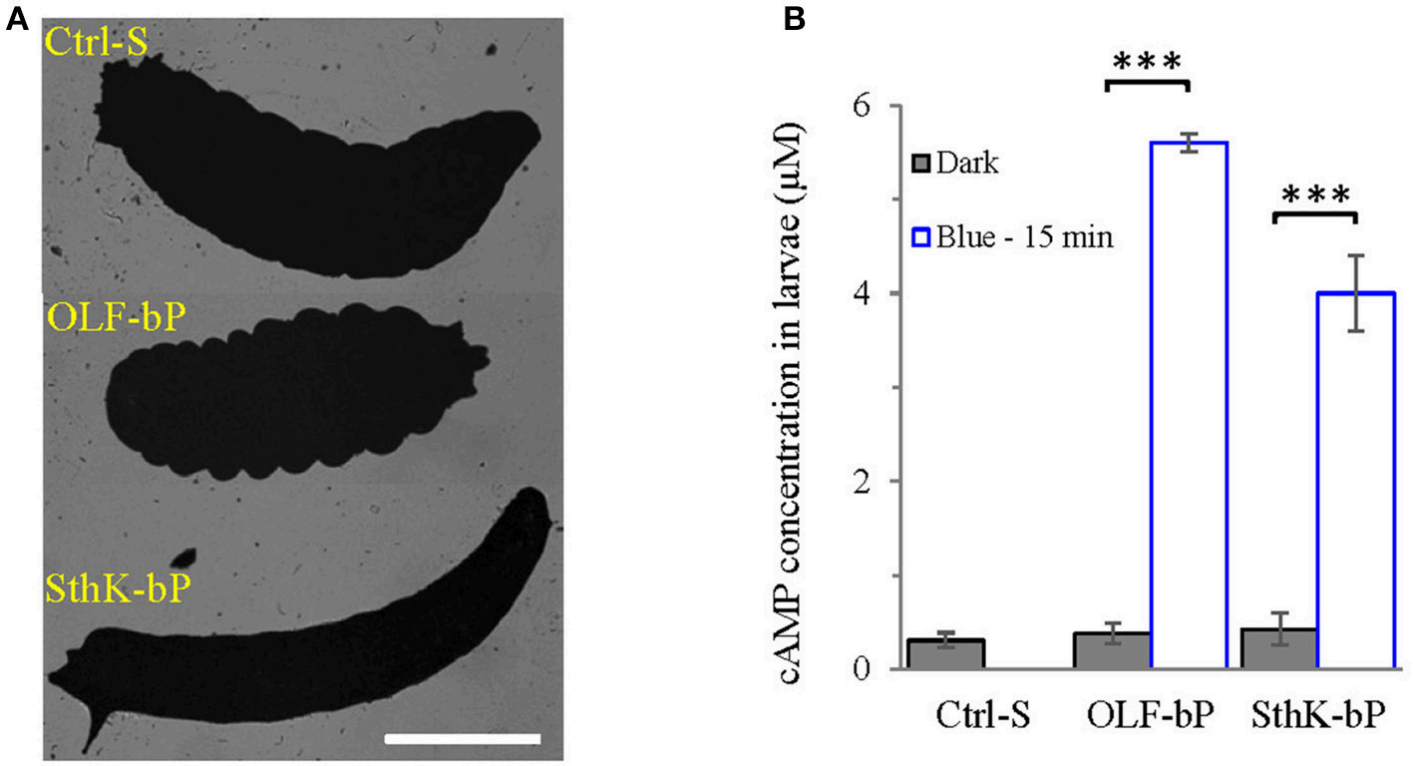
Functional expression of OLF-bP and SthK-bP in *Drosophila* larval motoneurons. **(A)** Expression of OLF-bP and SthK-bP in the larval motoneurons showed different phenotypes upon continuous blue light illumination, Ctrl-S = UAS-SthK-T-YFP-bPAC-Ex/+, Scale bar = 1 mm. **(B)** Light induced cAMP production in larvae expressing OLF-bP and SthK-bP in motoneurons, blue light (473 nm, 0.3 mW/mm^2^), *n* = 3 experiments, each with 6 larvae, error bars = SD. To calculate the final cAMP concentration in larvae, we assumed 1 larva has a volume of 2 μl.

The larvae expressing OLF-bP and SthK-bP in motoneurons were paralyzed upon continuous blue light illumination, with a clearly observable body contraction for OLF-bP and body extension for SthK-bP larvae (Figure 5A). These observations fit the known effects of increased Na^+^/Ca^2+^ and K^+^ permeability in *Drosophila* motoneurons: Depolarization and Ca^2+^ influx yield increased intracellular Ca^2+^, synaptic transmission and muscle contraction, whereas increased K^+^ permeability hyperpolarizes motoneurons, synaptic transmission is blocked and muscles relax.

Fifteen minute illumination with blue light (473 nm, 0.3 mW/mm^2^) induced a ~15-fold cAMP concentration increase (for the whole larva) for OLF-bP larvae and ~10-fold for SthK-bP (Figure 5B) demonstrating the light-dependent cyclase activity of these constructs in transgenic larvae.

**Figure 6:**
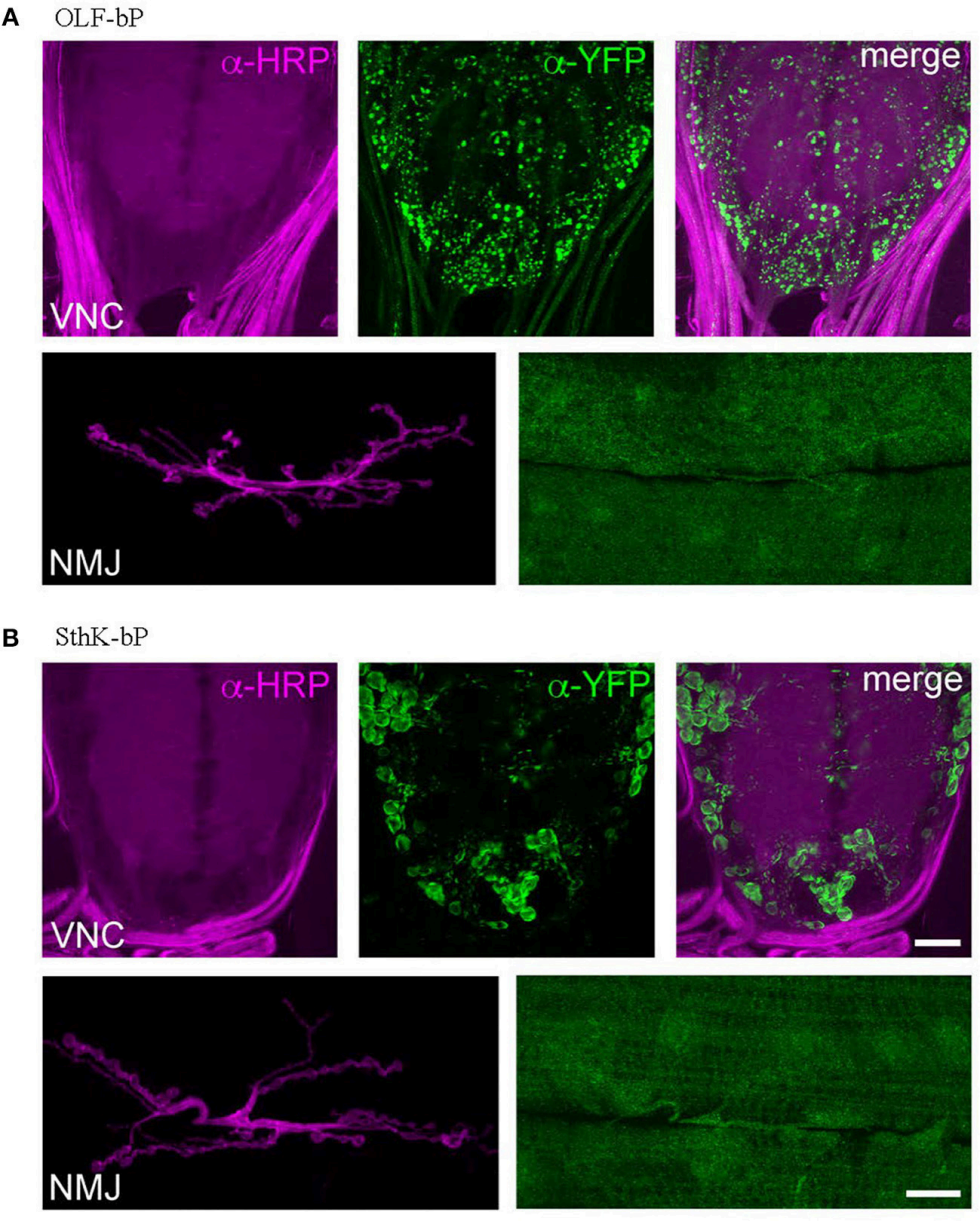
Expression of OLF-bP and SthK-bP in larval motoneurons. **(A)** Staining against YFP (green) reveals a clustered distribution of OLF-bP in motoneuron somata (larval VNC, upper panels), while no signal was detected at the NMJ (lower panels). Anti-HRP (horseradish peroxidase; magenta) was employed to stain neuronal membranes. **(B)** SthK-bP is enriched at cell body membranes in the VNC (upper panels). Similar to OLF-bP, no signal was detected at the NMJ (lower panels). Scale bars: 20 μm.

To investigate the distribution of OLF-bP and SthK-bP in motoneurons (ok6-GAL driver), immunohistochemistry of larval ventral nerve cords (VNCs) and neuromuscular junctions (NMJs) was performed (Figure 6). Both constructs show expression in motoneuron cell bodies of the VNC. While OLF-bP displays agglomeration within somata, SthK-bP localizes uniformly to cell body membranes. Neither construct could be detected in neuronal arborizations at the NMJ.

### Optogenetic control of larval motility

**Figure 7:**
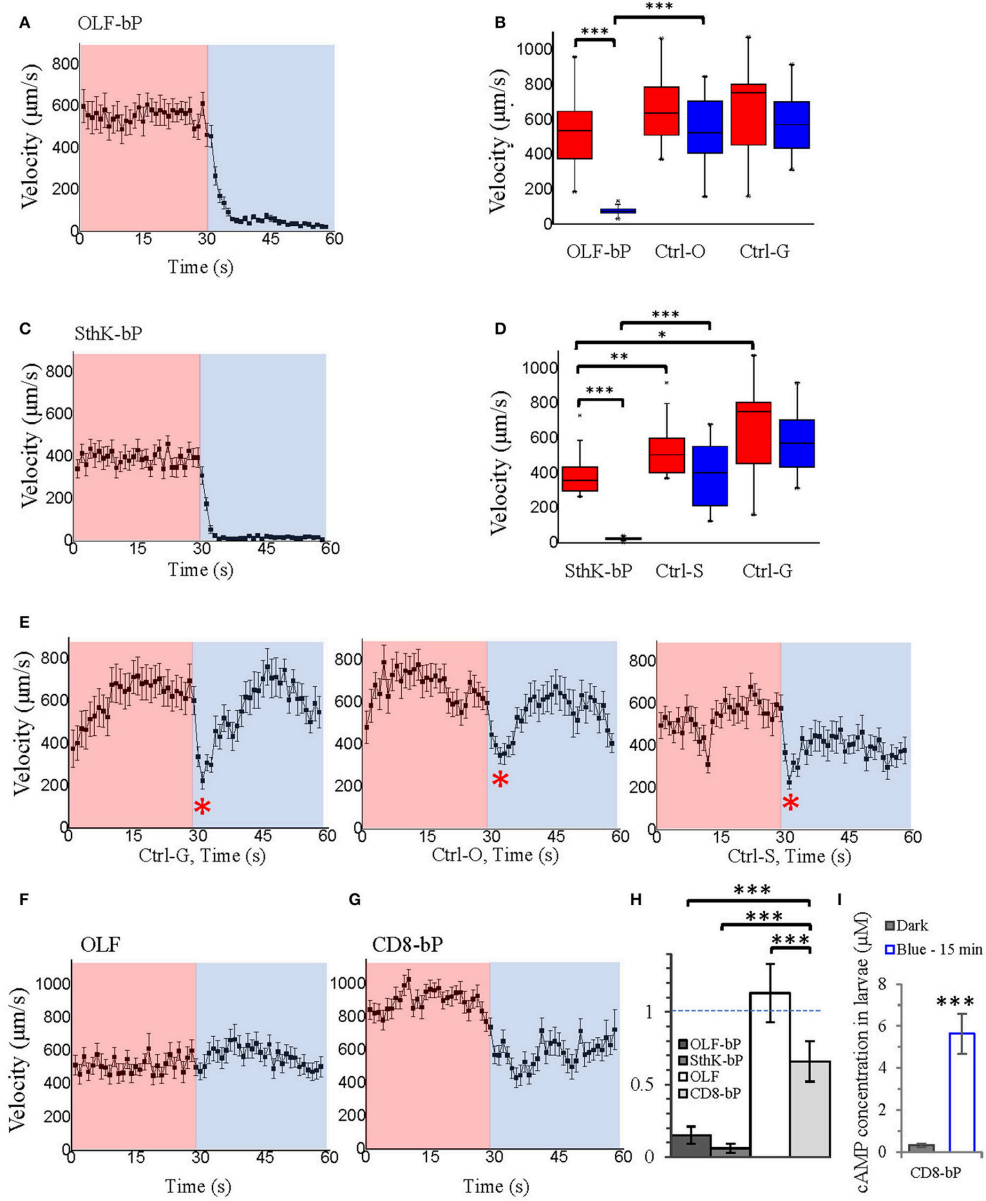
Optogenetic control of *Drosophila* larval motility. **(A)** Velocity of 3rd instar *Drosophila* larvae expressing OLF-bP in motoneurons under red and blue light. **(B)** Box plot of velocity data from OLF-bP expressing larvae and controls under red light and blue light conditions, Ctrl-G = +/OK-Gal4; Ctrl-O = UAS- OLF-T-YFP-bPAC-Ex/+. **(C)** Velocity of 3rd instar *Drosophila* larvae expressing SthK-bP in motoneurons under red and blue light. **(D)** Box plot of velocity data from Sthk-bP expressing larvae and controls under red light and blue light conditions. **(E)** Velocity of different *Drosophila* control larvae under red and blue light. For box plot graph, box line represents median, box edges represent 25 and 75 percentiles, whiskers represent 1 and 99 percentiles. **(F)** Velocity of D*rosophila* larvae expressing OLF-T-YFP-Ex (OLF) in motoneurons under control conditions (red) followed by 30 s of blue light illumination. **(G)** Velocity of D*rosophila* larvae expressing CD8-YFP-bPAC (CD8-bP) in motoneurons under control conditions followed by 30 s of blue light illumination. For A-G, n = 20 for each genotype; error bars = SEM. Red light (620 nm, 0.1 μW/cm^2^), blue light (470 nm, 1.6 mW/cm^2^). **(H)** Light to dark velocity ratios of different genotypes. *n* = 19, error bars = SD. **(I)** Light induced cAMP production of larvae expressing CD8-bP in motoneurons, blue light (473 nm, 0.3 mW/mm^2^). *n* = 3 experiments, each with six larvae, error bars = SD. To calculate the final cAMP concentration in larvae, we assumed that one larva has a volume of 2 μl.

Both OLF-bP and SthK-bP effectively paralyzed *Drosophila* larvae upon illumination with blue light (Videos 1, 2). To quantify the paralyzing effect, we recorded movements of larvae, expressing different constructs, and used the FIM (FTIR-based Imaging Method) tracking system to analyze and calculate posture- and motion-related parameters (Schmidt et al., 2014; Cosentino et al., 2015). As shown in Figure 7, Videos 3, 4, larvae expressing OLF-bP (Figures 7A,B) and SthK-bP (Figures 7C,D) in motoneurons were completely immobile during the 30 s blue light illumination (470 nm, 1.6 mW/cm^2^).

**Figure 8:**
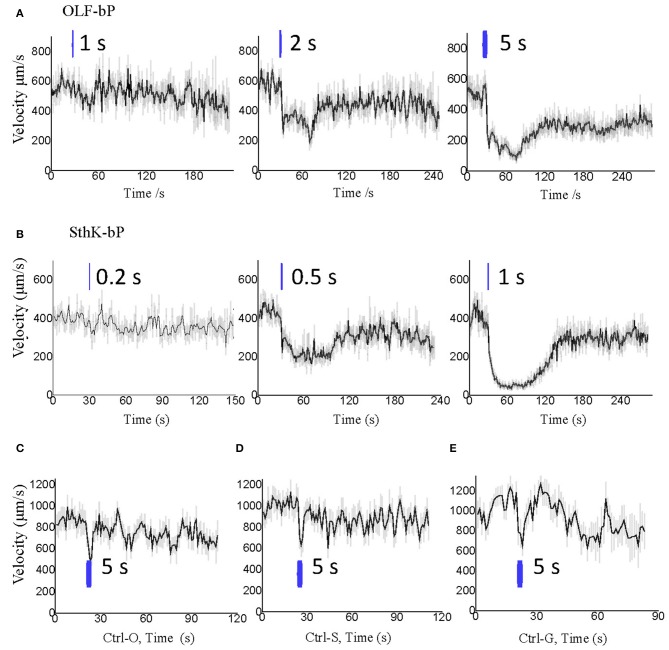
Optogenetic control of *Drosophila* larval motility with short illumination. **(A)** Velocity changes of 3rd instar *Drosophila* larvae expressing OLF-bP in motoneurons with 1, 2, and 5 s blue light illumination. **(B)** Velocity changes of 3rd instar *Drosophila* larvae expressing SthK-bP in motoneurons with 0.2, 0.5, and 1 s blue light illumination. **(C–E)** Velocity changes of 3 control lines with 5 s blue light illumination. For all, *n* = 20 for each genotype [except for **(C)**, here *n* = 16]; error bars (gray) = SEM. Control red light conditions (620 nm, 0.1 μW/cm^2^), blue light (470 nm 25 μW/cm^2^).

Larvae of the three control lines, UAS-OLF-T-YFP-bPAC-Ex/+ (Ctrl-O), UAS-SthK-T-YFP-bPAC-Ex/+ (Ctrl-S), and OK6-Gal4/+ (Ctrl-G), transiently slowed at the start of blue illumination but resumed their normal motion within seconds (Figure 7E, see asterisks). This transient response is a normal behavior of the larvae toward the changing light conditions. We also used shorter light pulses to see the effect of varying light dose on larval locomotion. SthK-bP larvae were more light-sensitive than OLF-bP larvae. The motility of OLF-bP larvae was obviously affected with 2 s (470 nm, 25 μW/cm^2^) illumination (Figure 8A) whereas SthK-bP larvae already showed a clear response to 0.5 s illumination (Figure 8B). The three controls only showed a mild and fast recovered natural response to 5 s illumination (Figure 8C). This difference in sensitivity between OLF-bP and SthK-bP correlates with their cAMP sensitivity. The cAMP EC_50_, for SthK is 3.7 ± 0.55 μM (Altenhofen et al., 1991), and for OLF/T537S is 14 ± 4 μM (Brams et al., 2014). Recovery from paralysis was also faster for shorter light pulses (Figure 8). Channel closing relies on diffusion and hydrolysis of cAMP, which will take longer when the intracellular cAMP reaches higher concentrations.

We also observed that even under red light, larvae expressing SthK-bP in motoneurons showed lower motility than control larvae (Figure 7D). This observation might indicate that endogenous cAMP partially activates SthK due to its high cAMP affinity (EC_50_ = 3.7 ± 0.55 μM).

**Figure 9:**
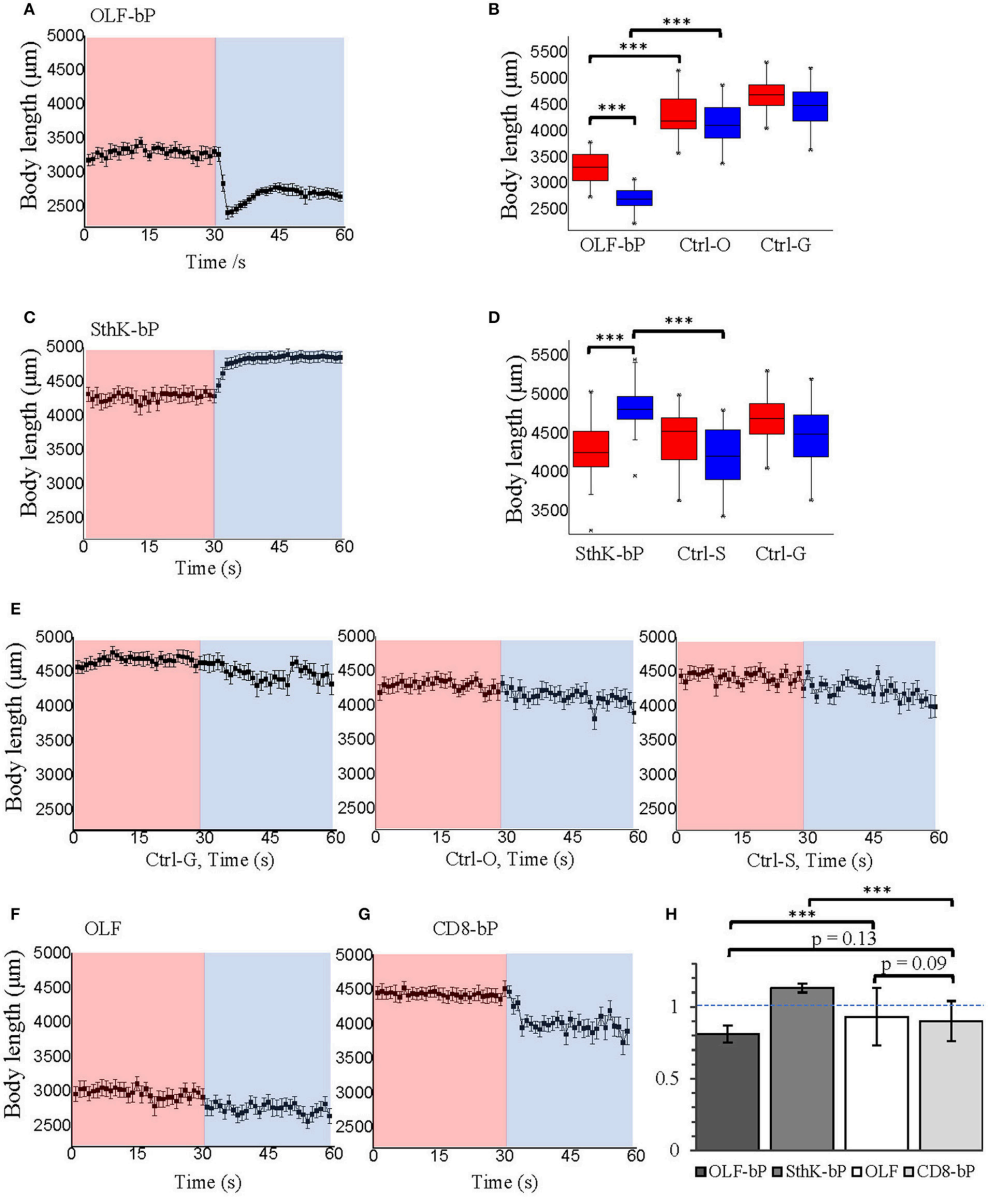
Optogenetic control of *Drosophila* larval body length with long illumination. **(A)** Body length of 3rd instar *Drosophila* larvae expressing OLF-bP in motoneurons under red and blue light. **(B)** Box plot of body length data from OLF-bP expressing larvae and controls under red light and blue light conditions. **(C)** Body length of 3rd instar *Drosophila* larvae expressing SthK-bP in motoneurons under red and blue light. **(D)** Box plot of body length data from Sthk-bP expressing larvae and controls under red light and blue light conditions. For box plot graph **(B,D)**, box line represents median, box edges represent 25 and 75 percentiles, whiskers represent 1 and 99 percentiles. **(E)** Body length of different *Drosophila* control larvae under red and blue light. Body length changes of 3rd instar *Drosophila* larvae expressing OLF **(F)** and CD8-bP **(G)** in motoneurons under control conditions followed by 30 s of blue light illumination. For **A–G**, *n* = 20 for each genotype; error bars = SEM. Red light (620 nm, 0.1 μW/cm^2^), blue light (470 nm, 1.6 mW/cm^2^). **(H)** Light to dark body length ratios of different genotypes. *n* = 19, error bars = SD.

As additional controls, we generated two further fly strains expressing either OLF or bPAC. Larvae expressing OLF alone [UAS-OLF-T-YFP-Ex/OK6-Gal4 (OLF)], showed no significant light response (Figure 7F). To create a membrane-bound bPAC control, we fused bPAC to the CD8 membrane anchor, which was previously used to target GFP to the plasma membrane in *Drosophila* (Lee and Luo, 1999). The OK6-Gal4/UAS-CD8-YFP-bPAC (CD8-bP) larvae showed a significant slowing of locomotion upon blue light illumination (470 nm, 1.6 mW/cm^2^) but did not become paralyzed in contrast to the larvae expressing either channel together with bPAC (Figure 7G). This is not surprising, as increased cAMP (via forskolin) is known to increase neurotransmitter release from larval motoneurons (Cheung et al., 2006), which could increase muscle tone and account for the changes in locomotion observed here. Indeed, body length decreased in these larvae when illuminated, indicative of muscle contraction (Figure 9G). The velocity of CD8-bP larvae dropped to 66% upon illumination, while OLF-bP larvae slowed to 15% and SthK-bP larvae to 6% (Figure 7H).

Fifteen minute blue light (473 nm, 0.3 mW/mm^2^) illumination generated an ~18-fold increase in cAMP for CD8-bP larvae (Figure 7I), which is similar to the bPAC activity of OLF-bP animals.

### Optogenetic control of *Drosophila* larval body length

While activation of OLF-bP and SthK-bP both paralyze the larvae, their respective phenotypes are opposing as already shown in Figure 5A, Video 1 and 2. OLF-bP led to body contraction and SthK-bP led to body extension. To quantify this opposing effect caused by OLF-bP and SthK-bP, we compared the impact on the larval body length upon illumination (Figure 9). While OLF-bP larvae contracted to around 80% of their previous body length (Figures 9A,B), SthK-bP larvae extended to around 116% (Figures 9C,D). This correlates well with the ion selectivity of the two CNG channels. OLF is a channel most permeable for Ca^2+^ and Na^+^, which would activate the motoneuron and lead to muscle contraction, while SthK is a K^+^-selective channel which would inhibit the motoneuron and lead to muscle relaxation. In contrast, control larvae did not change their body length in response to illumination, further confirming the channel-specific phenotype (Figure 9E). The influences of blue light illumination on body length were statistically significant for both OLF-bP and SthK-bP larvae while control larvae were not significantly different upon blue illumination (Figures 9B,D).

The channel-only-control, OLF in motoneurons, also showed no response to light regarding the body-length (Figure 9F). The membrane-bound bPAC control, CD8-bP in motoneurons, showed slight light-induced contraction (Figure 9G) possibly due to a cAMP-induced increase of neurotransmitter release from larval motoneurons (Cheung et al., 2006). Here, larval body length contracted to 90% upon illumination, while OLF-bP larvae contracted to 81% and SthK-bP larvae to 113% (Figure 9H).

In addition, for OLF-bP and the channel-only-control, OLF, both larvae and pupae were smaller (Figures 9B,F, for pupae see Supplementary Figures 2A,B), which suggests a probable effect of endogenous cAMP or cGMP on OLF and OLF-bP. It is worth mentioning here that the OLF/T537S mutant we are using here is very sensitive to cGMP with an EC_50_ of 0.7 ± 0.2 μM (Brams et al., 2014).

**Figure 10:**
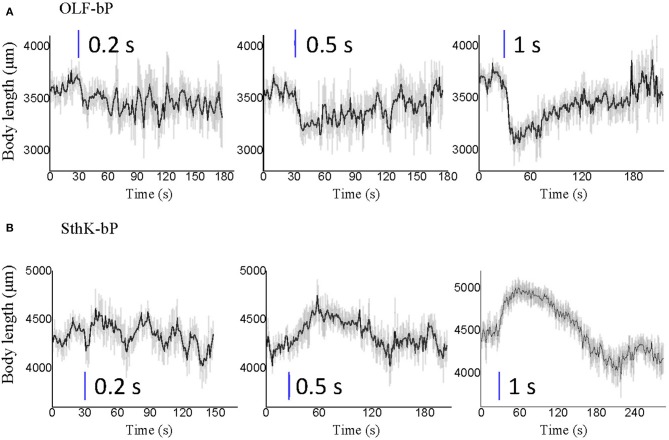
Optogenetic control of *Drosophila* larval body length with short illumination. Body length changes of 3rd instar *Drosophila* larvae expressing OLF-bP **(A)** and SthK-bP **(B)** in motoneurons with 0.2, 0.5, and 1 s blue light illumination. *n* = 20 for each genotype; error bars (gray) = SEM. Control conditions (red light 620 nm, 0.1 μW/cm^2^), blue light (470 nm, 25 μW/cm^2^).

Both the body contracting effect of OLF-bP and the body stretching effect of SthK-bP appeared to be fully reversible after blue light illumination, with a light-dose-dependent recovery time (Figure 10). Interestingly, both OLF-bP and SthK-bP larvae showed obvious body size changes, already with 0.5 s blue light illumination (470 nm, 25 μW/cm^2^).

## Discussion

We designed tools for light-activated Na^+^/Ca^2+^ and K^+^ permeability, combining bPAC and different CNG channels, optimized the fusion strategy and first tested their properties in *Xenopus* oocytes. The optimized fusion constructs were then shown to be effective at activating and inhibiting hippocampal neurons and were subsequently tested in *Drosophila* larval motoneurons with an easily interpretable readout (Pauls et al., 2015). Illumination led to body contraction with highly Ca^2+^ permeable OLF-bP, or extension with K^+^ selective SthK-bP. Both constructs paralyze the larvae effectively upon short light flashes.

There is high demand for an improved optogenetic inhibitory tool since the currently available inhibitory tools are not always effective. Whether ACRs hyperpolarize or depolarize cells depends on the intracellular Cl^−^ concentration (Mahn et al., 2016; Wiegert and Oertner, 2016). Hyperpolarizing light-activated pumps were shown to be able to inhibit action potentials (Zhang et al., 2007) but only at high light intensities and still with low turnover and efficiency. Sustained inhibition with proton pump-type tools may induce a pH-dependent Ca^2+^ influx and leads to increased spontaneous neurotransmitter release (Mahn et al., 2016). Hyperpolarization by halorhodopsin may also result in enhanced synaptic transmission (Mattingly et al., 2018). A powerful light activated K^+^ channel has therefore been high on many wish lists.

The new SthK-bP induced large outward K^+^ currents and effectively inhibited hippocampal neurons. Furthermore, based on the narrow activation spectrum in the blue range and high light sensitivity of bPAC, it should be possible to combine SthK-bP with red light-activated ChRs such as Chrimson (Klapoetke et al., 2014) for modulating activation and inhibition of one cell with two colors. Detailed characterization of the inhibition effect and further optimization of SthK-bP are necessary in the future. This should be focused on two aspects, modifying the cAMP affinity and improving targeting to axons and presynaptic compartments.

When employing such synthetic optogenetic tools, the effects of raising intracellular cAMP need to be taken into consideration. Cyclic AMP is an important second messenger that may itself induce physiological changes which could be cell type and developmental stage dependent. The new tools have been optimized to produce little cAMP very close to the channels to minimize bulk increases in cAMP. However, bPAC only controls are needed to discriminate between the effects of cAMP, Ca^2+^ and K^+^.

Endogenous cAMP and cGMP may also directly activate the channels independently of light-induced cAMP. This is a likely explanation for the smaller size of *Drosophila* larvae and pupae expressing OLF-bP and the channel alone. Larvae expressing SthK-bP in motoneurons, turned out to move slightly slower. This might also be due to activation of the channels by endogenous cAMP. We also observed that expression of OLF-bP and SthK-bP in motoneurons leads to eclosion failure limiting their use to larvae.

Several strategies can be applied to overcome this problem and to enable the application of these tools in adult flies. Firstly, an inducible gene expression strategy can be applied, such as temperature-sensitive gene expression using tubGAL80ts (McGuire et al., 2004). Secondly, it may be possible to develop OLF or SthK mutants with lower cAMP and cGMP sensitivities.

In summary, we have generated new and efficient tools for optogenetic manipulation of Ca^2+^ or K^+^ permeability. These tools work efficiently in *Xenopus* oocytes, *Drosophila* larvae and hippocampal cultures.

## Author contributions

SG and GN designed the molecular tools. SG and JY-S performed the experiments in *Xenopus* oocytes. SB carried out the *Drosophila* work with the help of DP, NE, RJK, and SG. OC performed experiments and analyzed data from hippocampal neurons and together with CG wrote parts of the manuscript. SG and GN wrote the first draft and all authors revised the paper and approved the final version to be published.

### Conflict of interest statement

The authors declare that the research was conducted in the absence of any commercial or financial relationships that could be construed as a potential conflict of interest.

## References

[B1] Airan R. D. Thompson K. R. Fenno L. E. Bernstein H. Deisseroth K. (2009). Temporally precise *in vivo* control of intracellular signalling. Nature 458, 1025–1029. 10.1038/nature07926 19295515

[B2] Altenhofen W. Ludwig J. Eismann E. Kraus W. Bonigk W. Kaupp U. (1991). B. Control of ligand specificity in cyclic nucleotide-gated channels from rod photoreceptors and olfactory epithelium. Proc. Natl. Acad. Sci. U. S. A. 88, 9868–9872. 10.1073/pnas.88.21.9868 1719541 PMC52822

[B3] Banghart M. Borges K. Isacoff E. Trauner D. Kramer R. (2004). H. Light-activated ion channels for remote control of neuronal firing. Nat. Neurosci. 7, 1381–1386. 10.1038/nn1356 15558062 PMC1447674

[B4] Bi A. D. Cui J. J. Ma Y. P. Olshevskaya E. Pu M. L. Dizhoor A. M.. (2006). Ectopic expression of a microbial-type rhodopsin restores visual responses in mice with photoreceptor degeneration. Neuron, 50, 23–33. 10.1016/j.neuron.2006.02.026 16600853 PMC1459045

[B5] Boyden E. S. Zhang F. Bamberg E. Nagel G. Deisseroth K. (2005). Millisecond-timescale, genetically targeted optical control of neural activity. Nat. Neurosc. 8, 1263–1268. 10.1038/nn1525 16116447

[B6] Brams M. Kusch J. Spurny R. Benndorf K. Ulens C. (2014). Family of prokaryote cyclic nucleotide-modulated ion channels. Proc. Natl. Acad. Sci. U. S. A. 111, 7855–7860. 10.1073/pnas.1401917111 24821777 PMC4040583

[B7] Cheung U. Atwood H. L. Zucker R. S. (2006). Presynaptic effectors contributing to cAMP-induced synaptic potentiation in Drosophila. J. Neurobiol. 66, 273–280. 10.1002/neu.20218 16329127

[B8] Chow B. Y. Han X. Dobry A. S. Qian X. Chuong A. S. Li M.. (2010). High-performance genetically targetable optical neural silencing by light-driven proton pumps. Nature 463, 98–102. 10.1038/nature08652 20054397 PMC2939492

[B9] Cosentino C. Alberio L. Gazzarrini S. Aquila M. Romano E. Cermenati S.. (2015). Optogenetics. Engineering of a light-gated potassium channel. Science 348, 707–710. 10.1126/science.aaa2787 25954011

[B10] Dawydow A. Gueta R. Ljaschenko D. Ullrich S. Hermann M. Ehmann N.. (2014). Channelrhodopsin-2-XXL, a powerful optogenetic tool for low-light applications. Proc. Natl. Acad. Sci. U.S.A. 111, 13972–7. 10.1073/pnas.1408269111 25201989 PMC4183338

[B11] Dzeja C. Hagen V. Kaupp U. B. Frings S. (1999). Ca^2+^ permeation in cyclic nucleotide-gated channels. Embo J. 18, 131–144. 10.1093/emboj/18.1.131 9878057 PMC1171109

[B12] Frings S. Seifert R. Godde M. Kaupp U. (1995). Profoundly different calcium permeation and blockage determine the specific function of distinct cyclic nucleotide-gated channels. Neuron 15, 169–179. 10.1016/0896-6273(95)90074-8 7542461

[B13] Gao S. Q. Nagpal J. Schneider M. W. Kozjak-Pavlovic V. Nagel G. Gottschalk A. (2015). Optogenetic manipulation of cGMP in cells and animals by the tightly light-regulated guanylyl-cyclase opsin CyclOp. Nat. Commun. 6:8046. 10.1038/ncomms9046 26345128 PMC4569695

[B14] Gee C. E. Ohmert I. Wiegert J. S. Oertner T. (2017). Preparation of slice cultures from rodent hippocampus. Cold Spring Harb. Protoc. 2017: pdb prot094888. 10.1101/pdb.prot094888 28148854

[B15] Govorunova E. G. Sineshchekov O. A. Janz R. Liu X. Q. Spudich J. (2015). Natural light-gated anion channels: A family of microbial rhodopsins for advanced optogenetics. Science 349, 647–650. 10.1126/science.aaa7484 26113638 PMC4764398

[B16] Gradinaru V. Zhang F. Ramakrishnan C. Mattis J. Prakash R. Diester I.. (2010). Molecular and cellular approaches for diversifying and extending optogenetics. Cell 141, 154–165 10.1016/j.cell.2010.02.037 20303157 PMC4160532

[B17] He L. Zhang Y. Ma G. Tan P. Li Z. Zang S.. (2015). Near-infrared photoactivatable control of Ca(2+) signaling and optogenetic immunomodulation. Elife 4:e10024. 10.7554/eLife.10024 26646180 PMC4737651

[B18] Iseki M. Matsunaga S. Murakami A. Ohno K. Shiga K. Yoshida K.. (2002). A blue-light-activated adenylyl cyclase mediates photoavoidance in Euglena gracilis. Nature 415, 1047–1051. 10.1038/4151047a 11875575

[B19] Ishizuka T. Kakuda M. Araki R. Yawo H. (2006). Kinetic evaluation of photosensitivity in genetically engineered neurons expressing green algae light-gated channels. Neurosci. Res. 54, 85–94. 10.1016/j.neures.2005.10.009 16298005

[B20] Kesters D. Brams M. Nys M. Wijckmans E. Spurny R. Voets T.. (2015). Structure of the SthK carboxy-terminal region reveals a gating mechanism for cyclic nucleotide-modulated ion channels. PLoS ONE 10:e0116369. 10.1371/journal.pone.0116369 25625648 PMC4308110

[B21] Klapoetke N. C. Murata Y. Kim S. S. Pulver S. R. Birdsey-Benson A. Cho Y. K. (2014). Independent optical excitation of distinct neural populations. Nat. Methods 11, 338–346. 10.1038/nmeth.2836 24509633 PMC3943671

[B22] Kleinlogel S. Feldbauer K. Dempski R. E. Fotis H. Wood P. G. Bamann C.. (2011). Ultra light-sensitive and fast neuronal activation with the Ca(2)+-permeable channelrhodopsin CatCh. Nat. Neurosci. 14, 513–518. 10.1038/nn.2776 21399632

[B23] Kyung T. Lee S. Kim J. E. Cho T. Park H. Jeong Y. M.. (2015). Optogenetic control of endogenous Ca(2+) channels *in vivo*. Nat. Biotechnol. 33, 1092–1096. 10.1038/nbt.3350 26368050

[B24] Lee T. Luo L. (1999). Mosaic analysis with a repressible cell marker for studies of gene function in neuronal morphogenesis. Neuron 22, 451–461. 10.1016/S0896-6273(00)80701-1 10197526

[B25] Li X. Gutierrez D. V. Hanson M. G. Han J. Mark M. D. Chiel H.. (2005). Fast noninvasive activation and inhibition of neural and network activity by vertebrate rhodopsin and green algae channelrhodopsin. Proc. Natl. Acad. Sci. U. S. A. 102, 17816–17821. 10.1073/pnas.0509030102 16306259 PMC1292990

[B26] Lin J. Y. Knutsen P. M. Muller A. Kleinfeld D. Tsien R. Y. (2013). ReaChR: a red-shifted variant of channelrhodopsin enables deep transcranial optogenetic excitation. Nat. Neurosci. 16, 1499–1508. 10.1038/nn.3502 23995068 PMC3793847

[B27] Ljaschenko D. Ehmann N. Kittel R. J. (2013). Hebbian plasticity guides maturation of glutamate receptor fields *in vivo*. Cell Rep. 3, 1407–1413. 10.1016/j.celrep.2013.04.003 23643532

[B28] Mahn M. Prigge M. Ron S. Levy R. Yizhar O. (2016). Biophysical constraints of optogenetic inhibition at presynaptic terminals. Nat. Neurosci. 19, 554–556. 10.1038/nn.4266 26950004 PMC4926958

[B29] Mattingly M. Weineck K. Costa J. Cooper R. L. (2018). Hyperpolarization by activation of halorhodopsin results in enhanced synaptic transmission: neuromuscular junction and CNS circuit. PLoS ONE 13:e0200107. 10.1371/journal.pone.0200107 29969493 PMC6029800

[B30] McGuire S. E. Mao Z. Davis R. L. (2004). Spatiotemporal gene expression targeting with the TARGET and gene-switch systems in Drosophila. Sci. STKE 2004:pl6. 10.1126/stke.2202004pl6 14970377

[B31] Nagel G. Brauner M. Liewald J. F. Adeishvili N. Bamberg E. Gottschalk A. (2005). Light activation of channelrhodopsin-2 in excitable cells of Caenorhabditis elegans triggers rapid behavioral responses. Curr. Biol. 15, 2279–2284. 10.1016/j.cub.2005.11.032 16360690

[B32] Nagel G. Ollig D. Fuhrmann M. Kateriya S. Mustl A. M. Bamberg E.. (2002). Channelrhodopsin-1: a light-gated proton channel in green algae. Science 296, 2395–2398. 10.1126/science.1072068 12089443

[B33] Nagel G. Szellas T. Huhn W. Kateriya S. Adeishvili N. Berthold P.. (2003). Channelrhodopsin-2, a directly light-gated cation-selective membrane channel. Proc. Natl. Acad. Sci. U. S. A. 100, 13940–13945. 10.1073/pnas.1936192100 14615590 PMC283525

[B34] Pauls D. von Essen A. Lyutova R. van Giesen L. Rosner R. Wegener C.. (2015). Potency of transgenic effectors for neurogenetic manipulation in *Drosophila* larvae. Genetics 199, 25–37. 10.1534/genetics.114.172023 25359929 PMC4286689

[B35] Risse B. Berh D. Otto N. Klambt C. Jiang X. (2017). FIMTrack: an open source tracking and locomotion analysis software for small animals. PLoS Comput. Biol. 13:e1005530. 10.1371/journal.pcbi.1005530 28493862 PMC5444858

[B36] Risse B. Thomas S. Otto N. Lopmeier T. Valkov D. Jiang X.. (2013). FIM, a novel FTIR-based imaging method for high throughput locomotion analysis. PLoS ONE 8:e53963. 10.1371/journal.pone.0053963 23349775 PMC3549958

[B37] Ryu M. H. Moskvin O. V. Siltberg-Liberles J. Gomelsky M. (2010). Natural and engineered photoactivated nucleotidyl cyclases for optogenetic applications. J. Biol. Chem. 285, 41501–41508. 10.1074/jbc.M110.177600 21030591 PMC3009876

[B38] Sanyal S. (2009). Genomic mapping and expression patterns of C380, OK6 and D42 enhancer trap lines in the larval nervous system of Drosophila. Gene. Expr. Patterns. 9, 371–380. 10.1016/j.gep.2009.01.002 19602393

[B39] Scheib U. Stehfest K. Gee C. E. Korschen H. G. Fudim R. Oertner T. G.. (2015). The rhodopsin-guanylyl cyclase of the aquatic fungus Blastocladiella emersonii enables fast optical control of cGMP signaling. Sci. Sign. 8:rs8. 10.1126/scisignal.aab0611 26268609

[B40] Schmidt D. Tillberg P. W. Chen F. Boyden E. S. (2014). A fully genetically encoded protein architecture for optical control of peptide ligand concentration. Nat. Commun. 5:3019. 10.1038/ncomms4019 24407101 PMC4035689

[B41] Scholz N. Guan C. Nieberler M. Grotemeyer A. Maiellaro I. Gao S.. (2017). Mechano-dependent signaling by Latrophilin/CIRL quenches cAMP in proprioceptive neurons. Elife 6:28360. 10.7554/eLife.28360 28784204 PMC5548486

[B42] Schröder-Lang S. Schwarzel M. Seifert R. Strunker T. Kateriya S. Looser J.. (2007). Fast manipulation of cellular cAMP level by light *in vivo*. Nat. Methods 4, 39–42. 10.1038/nmeth975 17128267

[B43] Stewart B. A. Atwood H. L. Renger J. J. Wang J. Wu C. F. (1994). Improved stability of Drosophila larval neuromuscular preparations in haemolymph-like physiological solutions. J. Comp. Physiol. A 175, 179–191. 10.1007/BF00215114 8071894

[B44] Stierl M. Stumpf P. Udwari D. Gueta R. Hagedorn R. Losi A.. (2011). Light modulation of cellular cAMP by a small bacterial photoactivated adenylyl cyclase, bPAC, of the soil bacterium beggiatoa. J. Biol. Chem. 286, 1181–1188. 10.1074/jbc.M110.185496 21030594 PMC3020725

[B45] Venken K. J. He Y. Hoskins R. A. Bellen H. J. (2006). P[acman]: a BAC transgenic platform for targeted insertion of large DNA fragments in D. melanogaster. Science 314, 1747–1751. 10.1126/science.1134426 17138868

[B46] Wiegert J. S. Gee C. E. Oertner T. G. (2017). Single-Cell Electroporation of Neurons. Cold Spring Harb. Protoc. 2017:pdb prot094904. 10.1101/pdb.prot094904 28148856

[B47] Wiegert J. S. Oertner T. G. (2016). How (not) to silence long-range projections with light. Nat. Neurosci. 19, 527–528. 10.1038/nn.4270 27021941

[B48] Zhang F. Wang L. P. Brauner M. Liewald J. F. Kay K. Watzke N.. (2007). Multimodal fast optical interrogation of neural circuitry. Nature 446, 633–639. 10.1038/nature05744 17410168

