# Supplementary material for: Synthetic Light-Activated Ion Channels for Optogenetic Activation and Inhibition
Source: Front Neurosci. 2018 Oct 2;12:643. doi: 10.3389/fnins.2018.00643 (PMC6176052; doi:10.3389/fnins.2018.00643)
Supplement: Supplementary file 1 [file Data_Sheet_1.PDF]

Supplementary Material

Data Sheet for DNA sequences of different constructs used in this study.

Olf-YFP

atgacag Olf--atggtg YFP

atgacagaaaaagccaatggcgtgaagagctccccagccaataaccacaaccaccatgcccctcctgccatcaaggccagtggcaa

agatgaccacagggccagcagccggccacagtctgctgctgctgatgacacctcctcagagctacagcaactggcagagatggatg

ccccccagcagaggaggggtggcttccgcaggattgcccgcctggtggggtcctcagagagtgggcttacaggaacttccgtgag

gaggagcctagacctgactcattccttgagcgtttccgggggcctgagctccacaccgtgacaacacaacaaggagacggcaaagg

cgacaaggacggcgagggcaagggcaccaagaagaagtttgaactctttgtcttggacccagccggggactggtactaccgctggc

tttttctcattgccttgcccgtcctctacaactggtgcctattggtggccagagcctgcttcagtgacctgcagaaaggctactacatagtg

tggctggtgctggattacgtctcagatgtggtctacatcgcagacctcttcatccgactgcgcacaggtttcttggagcaggggctactg

gtgaaagacaccaagaagttgcgggacaactacatccacaccatgcagtttaagctggatgtggcctccatcatccctacagacctgat

ctattttgctgtggggatccataaccctgaggtgcgcttcaaccgcctgctacactttgcccgcatgtttgagttctttgaccgcactgaga

cacgcaccagctaccccaacatcttccgaataagcaacctgatcctctacatcttgatcatcattcactggaatgcctgcatctactatgc

catctccaagtccatcggctttggggtagacacctgggtttaccccaacatcactgaccctgagtatggctacctgtctagggagtacat

ctattgcctttactggtctacactgaccctcaccaccattggggagacaccacccccctgtaaaggatgaggagtacctgtttgtcatctttg

acttcctgattggtgtcctcatctttgccaccatcgtgggaaatgtgggctccatgatctccaacatgaatgccacccgggctgagttcca

ggccaagattgatgctgtcaaacattatatgcagttccgaaaggtcagcaaggagatggaagccaaggtcattaggtggtttgactactt

gtggaccaataagaagagtgtagatgagcgagaagtcctcaaaaacctgccagcaaagctcagggctgagatagccatcaacgtcc

acctgtccacactcaagaaagtgcgcatctttcaggactgtgaggctggcctgctggtggaactggtattaaagctccggcctcaggtc

tttagccctggggactacatttgccgcaaggggggatattgggaaggagatgtacataatcaaggagggaaaattggcagtggtggctg

atgacggtgtcactcagtatgccctgctctcggctgggagttgctttggagagatcagtatccttaatattaagggcagcaaaatgggca

atcggcgcacagccaacatccgcagtcttggctactctgatctgttctgcttgtccaaggatgatcttatggaagctgtgactgagtaccc

tgatgccaagagggtcttggaggagagaggccgggagattctgatgaaggagggcttgttggatgagaatgaggtggcagccagca

tggaggtagatgtgcaggaaaagctagaacagctggagaccaacatggacaccttgtacactcgttttgcccgcctgctggccgagta

cacgggagcccagcagaagctcaagcagcgcatcacagttttggaaacgaagatgaagcagaataatgaggatgactccctgtcag

atgggatgaacagcccagagccacctgccgagaagccactcgagatggtgagcaagggcgaggagctgttcaccggggtggtgc

ccatcctggtcgagctggacggcgacgtaaacggccacaagttcagcgtgtccggcgagggcgagggcgatgccacctacggcaa

gctgaccctgaagttcatctgcaccaccggcaagctgcccgtgccctggcccaccctcgtgaccaccttcggctacggcctgcagtg

cttcgcccgctaccccgaccacatgaagcagcacgacttcttcaagtccgccatgcccgaaggctacgtccaggagcgcaccatcttc

ttcaaggacgacggcaactacaagacccgcgccgaggtgaagttcgagggcgacaccctggtgaaccgcatcgagctgaagggc
atcgacttcaaggaggacggcaacatcctggggcacaagctggagtacaactacaacagccacaacgtctatatcatggccgacaa
gcagaagaacggcatcaaggtgaacttcaagatccgccacaacatcgaggacggcagcgtgcagctcgccgaccactaccagcag
aacacccccatcggcgacggccccgtgctgctgcccgacaaccactacctgagctaccagtccgccctgagcaaagaccccaacg
agaagcgcgatcacatggtcctgctggagttcgtgaccgccgccgggatcactctcggcatggacgagctgtacaag**taa**

bPAC-YFP

atgatg bPAC--atggtg YFP

atgatgaagcggctggtgtacatcagcaagatcagcggccacctgagcctggaagagatccagcggatcggcaaggtgtccatcaa
gaacaaccagcgggacaacatcaccggcgtgctgctgtacctgcagggcctgttcttccagatcctggaaggcgagaacgagaagg
tggacaagctgtacaagaaaatcctggtggacgaccggcacaccaacatcctgtgcctgaaaaccgagtacgacatcaccgaccgg
atgttccccaactgggccatgaaaaccatcaacctgaacgagaacagcgagctgatgatccagcccatcaagagcctgctgcagacc
atcacccagagccaccgggtgctggaaaagtacatgcccgccagagtgatctacctgatcaaccagggcatcaacccccctgaccgt
ggagccccagctggtcgagaagatcatcttcttcagcgacatcctggccttcagcaccctgaccgagaagctgcccgtgaacgaggt
ggtcatcctggtcaaccggtacttcagcatctgcacccggatcatcagcgcctacggcggcgaagtgaccaagttcatcggcgactgc
gtgatggccagcttcaccaaagagcagggcgacgccgccatccggaccagcctggacatcatctccgagctgaagcagctgcggc
accacgtggaggccaccaacccccctgcacctgctgtacaccggcatcggcctgagctacggccacgtgatcgagggcaacatggg
cagcagcctgaagatggaccacaccctgctgggggacgccgtgaacgtggccgccaggctggaagccctgacaagacagctgcc
ctacgccctggcctttacagccggcgtgaagaagtgctgccaggcccagtggaccttcatcaacctgggagcccaccaggtcaagg
gcaagcaggaagccatcgaggtctacaccgtgaatgaggcccagaagtactacgacaccctgcagatcacccagctgatccggca
gaccctggaaaacgacaagccgcggacgtacgagcaaaagctgatctcggaggaggacctggcggccgccaccatggtgagcaa
gggcgaggagctgttcaccggggtggtgcccatcctggtcgagctggacggcgacgtaaacggccacaagttcagcgtgtccggc
gagggcgagggcgatgccacctacggcaagctgaccctgaagttcatctgcaccaccggcaagctgcccgtgccctggcccaccct
cgtgaccaccttcggctacggcctgcagtgcttcgcccgctaccccgaccacatgaagcagcacgacttcttcaagtccgccatgccc
gaaggctacgtccaggagcgcaccatcttcttcaaggacgacggcaactacaagacccgcgccgaggtgaagttcgagggcgaca
ccctggtgaaccgcatcgagctgaagggcatcgacttcaaggaggacggcaacatcctggggcacaagctggagtacaactacaac
agccacaacgtctatatcatggccgacaagcagaagaacggcatcaaggtgaacttcaagatccgccacaacatcgaggacggcag
cgtgcagctcgccgaccactaccagcagaacacccccatcggcgacggccccgtgctgctgcccgacaaccactacctgagctacc
agtccgccctgagcaaagaccccaacgagaagcgcgatcacatggtcctgctggagttcgtgaccgccgccgggatcactctcggc
atggacgagctgtacaag**taa**

Olf-bPAC

atgacag Olf-- atgatg bPAC

atgacagaaaaagccaatggcgtgaagagctccccagccaataaccacaaccaccatgcccctcctgccatcaaggccagtggcaa

agatgaccacagggccagcagccggccacagtctgctgctgctgatgacacctcctcagagctacagcaactggcagagatggatg

ccccccagcagaggaggggtggcttccgcaggattgcccgcctggtgggggtcctcagagagtgggcttacaggaacttccgtgag

gaggagcctagacctgactcattccttgagcgtttccgggggcctgagctccacaccgtgacaacacaacaaggagacggcaaagg

cgacaaggacggcgagggcaagggcaccaagaagaagtttgaactctttgtcttggacccagccggggactggtactaccgctggc

tttttctcattgccttgcccgtcctctacaactggtgcctattggtggccagagcctgcttcagtgacctgcagaaaggctactacatagtg

tggctggtgctggattacgtctcagatgtggtctacatcgcagacctcttcatccgactgcgcacaggtttcttggagcaggggctactg

gtgaaagacaccaagaagttgcgggacaactacatccacaccatgcagtttaagctggatgtggcctccatcatccctacagacctgat

ctattttgctgtggggatccataaccctgaggtgcgcttcaaccgcctgctacactttgcccgcatgtttgagttctttgaccgcactgaga

cacgcaccagctaccccaacatcttccgaataagcaacctgatcctctacatcttgatcatcattcactggaatgcctgcatctactatgc

catctccaagtccatcggctttggggtagacacctgggtttaccccaacatcactgaccctgagtatggctacctgtctagggagtacat

ctattgcctttactggtctacactgaccctcaccaccattggggagacaccaccccctgtaaaggatgaggagtacctgtttgtcatctttg

acttcctgattggtgtcctcatctttgccaccatcgtgggaaatgtgggctccatgatctccaacatgaatgccacccgggctgagttcca

ggccaagattgatgctgtcaaacattatatgcagttccgaaaggtcagcaaggagatggaagccaaggtcattaggtggtttgactactt

gtggaccaataagaagagtgtagatgagcgagaagtcctcaaaaacctgccagcaaagctcagggctgagatagccatcaacgtcc

acctgtccacactcaagaaagtgcgcatctttcaggactgtgaggctggcctgctggtggaactggtattaaagctccggcctcaggtc

tttagccctggggactacatttgccgcaagggggatattgggaaggagatgtacataatcaaggagggaaaattggcagtggtggctg

atgacggtgtcactcagtatgccctgctctcggctgggagttgctttggagagatcagtatccttaatattaagggcagcaaaatgggca

atcggcgcacagccaacatccgcagtcttggctactctgatctgttctgcttgtccaaggatgatcttatggaagctgtgactgagtaccc

tgatgccaagagggtcttggaggagagaggccgggagattctgatgaaggagggcttgttggatgagaatgaggtggcagccagca

tggaggtagatgtgcaggaaaagctagaacagctggagaccaacatggacaccttgtacactcgttttgcccgcctgctggccgagta

cacgggagcccagcagaagctcaagcagcgcatcacagttttggaaacgaagatgaagcagaataatgaggatgactccctgtcag

atgggatgaacagcccagagccacctgccgagaagccactcgagatgatgaagcggctggtgtacatcagcaagatcagcggcca

cctgagcctggaagagatccagcggatcggcaaggtgtccatcaagaacaaccagcgggacaacatcaccggcgtgctgctgtacc

tgcagggcctgttcttccagatcctggaaggcgagaacgagaaggtggacaagctgtacaagaaaatcctggtggacgaccggcac

accaacatcctgtgcctgaaaaccgagtacgacatcaccgaccggatgttccccaactgggccatgaaaaccatcaacctgaacgag

aacagcgagctgatgatccagcccatcaagagcctgctgcagaccatcacccagagccaccgggtgctggaaaagtacatgcccgc

cagagtgatctacctgatcaaccagggcatcaacccccctgaccgtggagccccagctggtcgagaagatcatcttcttcagcgacatc

ctggccttcagcaccctgaccgagaagctgcccgtgaacgaggtggtcatcctggtcaaccggtacttcagcatctgcacccggatca

tcagcgcctacggcggcgaagtgaccaagttcatcggcgactgcgtgatggccagcttcaccaaagagcagggcgacgccgccat

ccggaccagcctggacatcatctccgagctgaagcagctgcggcaccacgtggaggccaccaacccccctgcacctgctgtacaccg

gcatcggcctgagctacggccacgtgatcgagggcaacatgggcagcagcctgaagatggaccacaccctgctgggggacgccgt

gaacgtggccgccaggctggaagccctgacaagacagctgccctacgccctggcctttacagccggcgtgaagaagtgctgccag

gcccagtggaccttcatcaacctgggagcccaccaggtcaagggcaagcaggaagccatcgaggtctacaccgtgaatgaggccc

agaagtactacgacaccctgcagatcacccagctgatccggcagaccctggaaaacgacaagccgcggacgtacgagcaaaagct

gatctcggaggaggacctggcggccgccacc**taa**

Olf-YFP-bPAC

atgacag Olf--atggtg YFP--atgatg bPAC

atgacagaaaaagccaatggcgtgaagagctccccagccaataaccacaaccaccatgcccctcctgccatcaaggccagtggcaa

agatgaccacagggccagcagccggccacagtctgctgctgctgatgacacctcctcagagctacagcaactggcagagatggatg

ccccccagcagaggaggggtggcttccgcaggattgcccgcctggtggggtcctcagagagtgggcttacaggaacttccgtgag

gaggagcctagacctgactcattccttgagcgtttccgggggcctgagctccacaccgtgacaacacaacaaggagacggcaaagg

cgacaaggacggcgagggcaagggcaccaagaagaagtttgaactctttgtcttggacccagccggggactggtactaccgctggc

tttttctcattgccttgcccgtcctctacaactggtgcctattggtggccagagcctgcttcagtgacctgcagaaaggctactacatagtg

tggctggtgctggattacgtctcagatgtggtctacatcgcagacctcttcatccgactgcgcacaggtttcttggagcaggggctactg

gtgaaagacaccaagaagttgcgggacaactacatccacaccatgcagtttaagctggatgtggcctccatcatccctacagacctgat

ctattttgctgtggggatccataaccctgaggtgcgcttcaaccgcctgctacactttgcccgcatgtttgagttctttgaccgcactgaga

cacgcaccagctaccccaacatcttccgaataagcaacctgatcctctacatcttgatcatcattcactggaatgcctgcatctactatgc

catctccaagtccatcggctttggggtagacacctgggtttaccccaacatcactgaccctgagtatggctacctgtctagggagtacat

ctattgcctttactggtctacactgaccctcaccaccattggggagacaccaccccctgtaaaggatgaggagtacctgtttgtcatctttg

acttcctgattggtgtcctcatctttgccaccatcgtgggaaatgtgggctccatgatctccaacatgaatgccacccgggctgagttcca

ggccaagattgatgctgtcaaacattatatgcagttccgaaaggtcagcaaggagatggaagccaaggtcattaggtggtttgactactt

gtggaccaataagaagagtgtagatgagcgagaagtcctcaaaaacctgccagcaaagctcagggctgagatagccatcaacgtcc

acctgtccacactcaagaaagtgcgcatctttcaggactgtgaggctggcctgctggtggaactggtattaaagctccggcctcaggtc

tttagccctggggactacatttgccgcaaggggggatattgggaaggagatgtacataatcaaggagggaaaattggcagtggtggctg

atgacggtgtcactcagtatgccctgctctcggctgggagttgctttggagagatcagtatccttaatattaagggcagcaaaatgggca

atcggcgcacagccaacatccgcagtcttggctactctgatctgttctgcttgtccaaggatgatcttatggaagctgtgactgagtaccc

tgatgccaagagggtcttggaggagagaggccgggagattctgatgaaggagggcttgttggatgagaatgaggtggcagccagca

tggaggtagatgtgcaggaaaagctagaacagctggagaccaacatggacaccttgtacactcgttttgcccgcctgctggccgagta

cacgggagcccagcagaagctcaagcagcgcatcacagttttggaaacgaagatgaagcagaataatgaggatgactccctgtcag

atgggatgaacagcccagagccacctgccgagaagccactcgagatggtgagcaagggcgaggagctgttcaccggggtggtgc

ccatcctggtcgagctggacggcgacgtaaacggccacaagttcagcgtgtccggcgagggcgagggcgatgccacctacggcaa

gctgaccctgaagctgatctgcaccaccggcaagctgcccgtgccctggcccaccctcgtgaccaccctgggctacggcctgcagt

gcttcgcccgctaccccgaccacatgaagcagcacgacttcttcaagtccgccatgcccgaaggctacgtccaggagcgcaccatct

tcttcaaggacgacggcaactacaagacccgcgccgaggtgaagttcgagggcgacaccctggtgaaccgcatcgagctgaaggg

catcgacttcaaggaggacggcaacatcctggggcacaagctggagtacaactacaacagccacaacgtctatatcaccgccgaca

agcagaagaacggcatcaaggccaacttcaagatccgccacaacatcgaggacggcggcgtgcagctcgccgaccactaccagc

agaacacccccatcggcgacggccccgtgctgctgcccgacaaccactacctgagctaccagtccgccctgagcaaagaccccaa

cgagaagcgcgatcacatggtcctgctggagttcgtgaccgccgccgggatcactctcggcatggacgagctgtacaagggtagcg

gtgggctgaggcttaataagatgaagcggctggtgtacatcagcaagatcagcggccacctgagcctggaagagatccagcggatc

ggcaaggtgtccatcaagaacaaccagcgggacaacatcaccggcgtgctgctgtacctgcagggcctgttcttccagatcctggaa

ggcgagaacgagaaggtggacaagctgtacaagaaaatcctggtggacgaccggcacaccaacatcctgtgcctgaaaaccgagt

acgacatcaccgaccggatgttccccaactgggccatgaaaaccatcaacctgaacgagaacagcgagctgatgatccagcccatc

aagagcctgctgcagaccatcacccagagccaccgggtgctggaaaagtacatgcccgccagagtgatctacctgatcaaccaggg

catcaacccccctgaccgtggagccccagctggtcgagaagatcatcttcttcagcgacatcctggccttcagcaccctgaccgagaag

ctgcccgtgaacgaggtggtcatcctggtcaaccggtacttcagcatctgcacccggatcatcagcgcctacggcggcgaagtgacc

aagttcatcggcgactgcgtgatggccagcttcaccaaagagcagggcgacgccgccatccggaccagcctggacatcatctccga

gctgaagcagctgcggcaccacgtggaggccaccaacccccctgcacctgctgtacaccggcatcggcctgagctacggccacgtg

atcgagggcaacatgggcagcagcctgaagatggaccacaccctgctgggggacgccgtgaacgtggccgccaggctggaagcc

ctgacaagacagctgccctacgccctggcctttacagccggcgtgaagaagtgctgccaggcccagtggaccttcatcaacctggga

gcccaccaggtcaagggcaagcaggaagccatcgaggtctacaccgtgaatgaggcccagaagtactacgacaccctgcagatca

cccagctgatccgacagaccctggaaaacgacaagccgcggacgtacgagcaaaagctgatctcggaggaggacctg**taa**

Olf-T-YFP-bPAC-Ex

atgacag Olf--aaatcc T--atggtg YFP--atgatg bPAC--ttttgc Ex

atgacagaaaaagccaatggcgtgaagagctccccagccaataaccacaaccaccatgcccctcctgccatcaaggccagtggcaa

agatgaccacagggccagcagccggccacagtctgctgctgctgatgacacctcctcagagctacagcaactggcagagatggatg

ccccccagcagaggaggggtggcttccgcaggattgcccgcctggtggggtcctcagagagtgggcttacaggaacttccgtgag

gaggagcctagacctgactcattccttgagcgtttccgggggcctgagctccacaccgtgacaacacaacaaggagacggcaaagg

cgacaaggacggcgagggcaagggcaccaagaagaagtttgaactctttgtcttggacccagccggggactggtactaccgctggc

tttttctcattgccttgcccgtcctctacaactggtgcctattggtggccagagcctgcttcagtgacctgcagaaaggctactacatagtg
tggctggtgctggattacgtctcagatgtggtctacatcgcagacctcttcatccgactgcgcacaggtttcttggagcagggggctactg
gtgaaagacaccaagaagttgcgggacaactacatccacaccatgcagtttaagctggatgtggcctccatcatccctacagacctgat
ctattttgctgtggggatccataaccctgaggtgcgcttcaaccgcctgctacactttgcccgcatgtttgagttctttgaccgcactgaga
cacgcaccagctaccccaacatcttccgaataagcaacctgatcctctacatcttgatcatcattcactggaatgcctgcatctactatgc
catctccaagtccatcggctttggggtagacacctgggtttaccccaacatcactgaccctgagtatggctacctgtctagggagtacat
ctattgcctttactggtctacactgaccctcaccaccattggggagacaccacccccctgtaaaggatgaggagtacctgtttgtcatctttg
acttcctgattggtgtcctcatctttgccaccatcgtgggaaatgtgggctccatgatctccaacatgaatgccacccgggctgagttcca
ggccaagattgatgctgtcaaacattatatgcagttccgaaaggtcagcaaggagatggaagccaaggtcattaggtggtttgactactt
gtggaccaataagaagagtgtagatgagcgagaagtcctcaaaaacctgccagcaaagctcagggctgagatagccatcaacgtcc
acctgtccacactcaagaaagtgcgcatctttcaggactgtgaggctggcctgctggtggaactggtattaaagctccggcctcaggtc
tttagccctggggactacatttgccgcaagggggatattgggaaggagatgtacataatcaaggagggaaaattggcagtggtggctg
atgacggtgtcactcagtatgccctgctctcggctgggagttgctttggagagatcagtatccttaatattaagggcagcaaaatgggca
atcggcggtccgccaacatccgcagtcttggctactctgatctgttctgcttgtccaaggatgatcttatggaagctgtgactgagtaccc
tgatgccaagagggtcttggaggagagaggccgggagattctgatgaaggagggcttgttggatgagaatgaggtggcagccagca
tggaggtagatgtgcaggaaaagctagaacagctggagaccaacatggacaccttgtacactcgttttgcccgcctgctggccgagta
cacgggagcccagcagaagctcaagcagcgcatcacagttttggaaacgaagatgaagcagaataatgaggatgactccctgtcag
atgggatgaacagcccagagccacctgccgagaagccactcgagaaatccagaattacttctgaaggggagtatatccctctggatc
aaatagacatcaatgttgtcgacactagtgggcttaatatggtgagcaagggcgaggagctgttcaccggggtggtgcccatcctggt
cgagctggacggcgacgtaaacggccacaagttcagcgtgtccggcgagggcgagggcgatgccacctacggcaagctgaccct
gaagctgatctgcaccaccggcaagctgcccgtgccctggcccaccctcgtgaccaccctgggctacggcctgcagtgcttcgccc
gctaccccgaccacatgaagcagcacgacttcttcaagtccgccatgcccgaaggctacgtccaggagcgcaccatcttcttcaagg
acgacggcaactacaagacccgcgccgaggtgaagttcgagggcgacaccctggtgaaccgcatcgagctgaagggcatcgactt
caaggaggacggcaacatcctggggcacaagctggagtacaactacaacagccacaacgtctatatcaccgccgacaagcagaag
aacggcatcaaggccaacttcaagatccgccacaacatcgaggacggcggcgtgcagctcgccgaccactaccagcagaacaccc
ccatcggcgacggccccgtgctgctgcccgacaaccactacctgagctaccagtccgccctgagcaaagaccccaacgagaagcg
cgatcacatggtcctgctggagttcgtgaccgccgccgggatcactctcggcatggacgagctgtacaagggtagcggtgggctgag
gcttaataagatgaagcggctggtgtacatcagcaagatcagcggccacctgagcctggaagagatccagcggatcggcaaggtgt
ccatcaagaacaaccagcgggacaacatcaccggcgtgctgctgtacctgcagggcctgttcttccagatcctggaaggcgagaac
gagaaggtggacaagctgtacaagaaaatcctggtggacgaccggcacaccaacatcctgtgcctgaaaaccgagtacgacatcac

cgaccggatgttccccaactgggccatgaaaaccatcaacctgaacgagaacagcgagctgatgatccagcccatcaagagcctgct

gcagaccatcacccagagccaccgggtgctggaaaagtacatgcccgccagagtgatctacctgatcaaccagggcatcaacccc

tgaccgtggagccccagctggtcgagaagatcatcttcttcagcgacatcctggccttcagcaccctgaccgagaagctgcccgtgaa

cgaggtggtcatcctggtcaaccggtacttcagcatctgcacccggatcatcagcgcctacggcggcgaagtgaccaagttcatcgg

cgactgcgtgatggccagcttcaccaaagagcagggcgacgccgccatccggaccagcctggacatcatctccgagctgaagcag

ctgcggcaccacgtggaggccaccaacccccctgcacctgctgtacaccggcatcggcctgagctacggccacgtgatcgagggca

acatgggcagcagcctgaagatggaccacaccctgctgggggacgccgtgaacgtggccgccaggctggaagccctgacaagac

agctgccctacgccctggcctttacagccggcgtgaagaagtgctgccaggcccagtggaccttcatcaacctgggagcccaccag

gtcaagggcaagcaggaagccatcgaggtctacaccgtgaatgaggcccagaagtactacgacaccctgcagatcacccagctgat

ccggcagaccctggaaaacgacaagccgcggacgtacgagcaaaagctgatctcggaggaggacctggcggccgccacctctag

attttgctatgaaaatgaagtt**taa**

SthK-T-YFP-bPAC-Ex

atgaag SthK--aaatcc T--atggtg YFP--atgatg bPAC--ttttgc Ex

atgaagtccagcgcctttagccaccccacctacaccctcgtgtggaaagtgggcatcctggccgtgaccctgtactacgccatcagaa

tcccccctgaccctggtgttccccagcctgttctctcccctgctgcccctggatatcctggcctctctggccctgatcgccgacatccctct

ggacttcgccttcgagagcagaaagaccagcggccggaagcctacactgctggcccctagcagactgcccgatctgctggctgctct

gcctctggacctgctggtgttcgccctgcatctgcctagccctctgagcctgctgtctctcgtgcggctgctgaagctgatctccgtgca

gagaagcgccacaagaatcctgagctaccggatcaaccccgccctgctgagactgctgagcctcgtgggctttatcctgctggccgc

ccacggaatcgcctgcggatggatgtctctgcagcccctagcgagtctcctgccggcacaagatacctgagcgccttctactggacc

atcaccaccctgaccaccatcggctacggcgacatcacccccagcacccctatccagaccgtgtacaccatcgtgatcgagctgctg

ggagccgctatgtacggcctcgtgatcggcaatatcgccagcctggtgtccaagctggacgccgccaaactgctgcaccgcgagcg

gatggaaagagtgaccgccttcctgtcctacaagaagatcagccccgagctgcagcggagaatcctggaatacttcgactacctgtgg

gagacaagacggggctacgaggaacgcgaggtgctgaaagagctgccccacccactgagactggccgtggccatggaaatccac

ggcgacgtgatcgaaaaggtgcccctgttcaagggcgctggcgaggacttcatccgggacatcatcctgcacctggaacccgtgatc

tacggccctggcgagtacatcatcagagccggcgagctgggcagcgacgtgtacttcatcaaccggggcagcgtggaagtgctgag

cgccgacgagaaaaccagatacgccatcctgagcgagggccagttcttcggcgagatggctctgatcctgagagcccccagaaccg

ccacagtgcgggccagaaccttctgcgacctgtaccggctggacaaagagacattcgacagaatcctgtcccgctaccccgagatcg

ccgcccagattcaggaactggctgtgcggcggaaagaggaactggaaggcggcaccagcagacggggcctcgagaaatccaga

attacttctgaaggggagtatatccctctggatcaaatagacatcaatgttgtcgacactagtgggcttaatatggtgagcaagggcgag

gagctgttcaccggggtggtgcccatcctggtcgagctggacggcgacgtaaacggccacaagttcagcgtgtccggcgagggcg

agggcgatgccacctacggcaagctgaccctgaagctgatctgcaccaccggcaagctgcccgtgccctggcccaccctcgtgacc

accctgggctacggcctgcagtgcttcgcccgctaccccgaccacatgaagcagcacgacttcttcaagtccgccatgcccgaaggc

tacgtccaggagcgcaccatcttcttcaaggacgacggcaactacaagacccgcgccgaggtgaagttcgagggcgacaccctggt

gaaccgcatcgagctgaagggcatcgacttcaaggaggacggcaacatcctggggcacaagctggagtacaactacaacagccac

aacgtctatatcaccgccgacaagcagaagaacggcatcaaggccaacttcaagatccgccacaacatcgaggacggcggcgtgc

agctcgccgaccactaccagcagaacacccccatcggcgacggccccgtgctgctgcccgacaaccactacctgagctaccagtcc

gccctgagcaaagaccccaacgagaagcgcgatcacatggtcctgctggagttcgtgaccgccgccgggatcactctcggcatgga

cgagctgtacaagggtagcggtgggctgaggcttaataagatgaagcggctggtgtacatcagcaagatcagcggccacctgagcc

tggaagagatccagcggatcggcaaggtgtccatcaagaacaaccagcgggacaacatcaccggcgtgctgctgtacctgcaggg

cctgttcttccagatcctggaaggcgagaacgagaaggtggacaagctgtacaagaaaatcctggtggacgaccggcacaccaaca

tcctgtgcctgaaaaccgagtacgacatcaccgaccggatgttccccaactgggccatgaaaaccatcaacctgaacgagaacagcg

agctgatgatccagcccatcaagagcctgctgcagaccatcacccagagccaccgggtgctggaaaagtacatgcccgccagagtg

atctacctgatcaaccagggcatcaacccccctgaccgtggagccccagctggtcgagaagatcatcttcttcagcgacatcctggcctt

cagcaccctgaccgagaagctgcccgtgaacgaggtggtcatcctggtcaaccggtacttcagcatctgcacccggatcatcagcgc

ctacggcggcgaagtgaccaagttcatcggcgactgcgtgatggccagcttcaccaaagagcagggcgacgccgccatccggacc

agcctggacatcatctccgagctgaagcagctgcggcaccacgtggaggccaccaacccccctgcacctgctgtacaccggcatcgg

cctgagctacggccacgtgatcgagggcaacatgggcagcagcctgaagatggaccacaccctgctgggggacgccgtgaacgtg

gccgccaggctggaagccctgacaagacagctgccctacgccctggcctttacagccggcgtgaagaagtgctgccaggcccagt

ggaccttcatcaacctgggagcccaccaggtcaagggcaagcaggaagccatcgaggtctacaccgtgaatgaggcccagaagta

ctacgacaccctgcagatcacccagctgatccggcagaccctggaaaacgacaagccgcggacgtacgagcaaaagctgatctcg

gaggaggacctggcggccgccacctctagattttgctatgaaaatgaagttt**taa**

CD8-YFP-bPAC

atggcc CD8--atggtg YFP--atgatg bPAC

atggcctcaccgttgacccgctttctgtcgctgaacctgctgctgctgggtgagtcgattatcctggggagtggagaagctaagccaca

ggcacccgaactccgaatctttccaaagaaaatggacgccgaacttggtcagaaggtggacctggtatgtgaagtgttggggtccgttt

cgcaaggatgctcttggctcttccagaactccagctccaaactcccccagcccaccttcgttgtctatatggcttcatcccacaacaagat

aacgtgggacgagaagctgaattcgtcgaaactgttttctgccatgagggacacgaataataagtacgttctcaccctgaacaagttca

gcaaggaaaacgaaggctactatttctgctcagtcatcagcaactcggtgatgtacttcagttctgtcgtgccagtccttcagaaagtgaa

ctctactactaccaagccagtgctgcgaactccctcacctgtgcaccctaccgggacatctcagccccagagaccagaagattgtcgg

ccccgtggctcagtgaaggggaccggattggacttcgcctgtgatatttacatctgggcacccttggccggaatctgcgtggcccttct

gctgtccttgatcatcactctcatctgctaccactccggaggcggaggccccctcgagatggtgagcaagggcgaggagctgttcacc

ggggtggtgcccatcctggtcgagctggacggcgacgtaaacggccacaagttcagcgtgtccggcgagggcgagggcgatgcc

acctacggcaagctgaccctgaagctgatctgcaccaccggcaagctgcccgtgccctggcccaccctcgtgaccaccctgggcta

cggcctgcagtgcttcgcccgctaccccgaccacatgaagcagcacgacttcttcaagtccgccatgcccgaaggctacgtccagga

gcgcaccatcttcttcaaggacgacggcaactacaagacccgcgccgaggtgaagttcgagggcgacaccctggtgaaccgcatcg

agctgaagggcatcgacttcaaggaggacggcaacatcctggggcacaagctggagtacaactacaacagccacaacgtctatatc

accgccgacaagcagaagaacggcatcaaggccaacttcaagatccgccacaacatcgaggacggcggcgtgcagctcgccgac

cactaccagcagaacacccccatcggcgacggccccgtgctgctgcccgacaaccactacctgagctaccagtccgccctgagcaa

agaccccaacgagaagcgcgatcacatggtcctgctggagttcgtgaccgccgccgggatcactctcggcatggacgagctgtaca

agggtagcggtgggctgaggcttaataagatgaagcggctggtgtacatcagcaagatcagcggccacctgagcctggaagagatc

cagcggatcggcaaggtgtccatcaagaacaaccagcgggacaacatcaccggcgtgctgctgtacctgcagggcctgttcttccag

atcctggaaggcgagaacgagaaggtggacaagctgtacaagaaaatcctggtggacgaccggcacaccaacatcctgtgcctgaa

aaccgagtacgacatcaccgaccggatgttccccaactgggccatgaaaaccatcaacctgaacgagaacagcgagctgatgatcc

agcccatcaagagcctgctgcagaccatcacccagagccaccgggtgctggaaaagtacatgcccgccagagtgatctacctgatc

aaccagggcatcaaccccctgaccgtggagccccagctggtcgagaagatcatcttcttcagcgacatcctggccttcagcaccctga

ccgagaagctgcccgtgaacgaggtggtcatcctggtcaaccggtacttcagcatctgcacccggatcatcagcgcctacggcggcg

aagtgaccaagttcatcggcgactgcgtgatggccagcttcaccaaagagcagggcgacgccgccatccggaccagcctggacatc

atctccgagctgaagcagctgcggcaccacgtggaggccaccaacccccctgcacctgctgtacaccggcatcggcctgagctacgg

ccacgtgatcgagggcaacatgggcagcagcctgaagatggaccacaccctgctgggggacgccgtgaacgtggccgccaggct

ggaagccctgacaagacagctgccctacgccctggcctttacagccggcgtgaagaagtgctgccaggcccagtggaccttcatca

acctgggagcccaccaggtcaagggcaagcaggaagccatcgaggtctacaccgtgaatgaggcccagaagtactacgacaccct

gcagatcacccagctgatccgacagaccctggaaaacgacaagccgcggacgtacgagcaaaagctgatctcggaggaggacct

g**taa**

Olf-T-YFP-Ex

atgacag Olf--aaatcc T--atggtg YFP--ttttgc Ex

atgacagaaaaagccaatggcgtgaagagctccccagccaataaccacaaccaccatgcccctcctgccatcaaggccagtggcaa

agatgaccacagggccagcagccggccacagtctgctgctgctgatgacacctcctcagagctacagcaactggcagagatggatg

ccccccagcagaggaggggtggcttccgcaggattgcccgcctggtggggtcctcagagagtgggcttacaggaacttccgtgag

gaggagcctagacctgactcattccttgagcgtttccgggggcctgagctccacaccgtgacaacacaacaaggagacggcaaagg

cgacaaggacggcgagggcaagggcaccaagaagaagtttgaactctttgtcttggacccagccggggactggtactaccgctggc

tttttctcattgccttgcccgtcctctacaactggtgcctattggtggccagagcctgcttcagtgacctgcagaaaggctactacatagtg

tggctggtgctggattacgtctcagatgtggtctacatcgcagacctcttcatccgactgcgcacaggtttcttggagcaggggctactg

gtgaaagacaccaagaagttgcgggacaactacatccacaccatgcagtttaagctggatgtggcctccatcatccctacagacctgat

ctattttgctgtggggatccataaccctgaggtgcgcttcaaccgcctgctacactttgcccgcatgtttgagttctttgaccgcactgaga

cacgcaccagctaccccaacatcttccgaataagcaacctgatcctctacatcttgatcatcattcactggaatgcctgcatctactatgc

catctccaagtccatcggctttggggtagacacctgggtttaccccaacatcactgaccctgagtatggctacctgtctagggagtacat

ctattgcctttactggtctacactgaccctcaccaccattggggagacaccaccccctgtaaaggatgaggagtacctgtttgtcatctttg

acttcctgattggtgtcctcatctttgccaccatcgtgggaaatgtgggctccatgatctccaacatgaatgccacccgggctgagttcca

ggccaagattgatgctgtcaaacattatatgcagttccgaaaggtcagcaaggagatggaagccaaggtcattaggtggtttgactactt

gtggaccaataagaagagtgtagatgagcgagaagtcctcaaaaacctgccagcaaagctcagggctgagatagccatcaacgtcc

acctgtccacactcaagaaagtgcgcatctttcaggactgtgaggctggcctgctggtggaactggtattaaagctccggcctcaggtc

tttagccctggggactacatttgccgcaagggggatattgggaaggagatgtacataatcaaggagggaaaattggcagtggtggctg

atgacggtgtcactcagtatgccctgctctcggctgggagttgctttggagagatcagtatccttaatattaagggcagcaaaatgggca

atcggcggtccgccaacatccgcagtcttggctactctgatctgttctgcttgtccaaggatgatcttatggaagctgtgactgagtaccc

tgatgccaagagggtcttggaggagagaggccgggagattctgatgaaggagggcttgttggatgagaatgaggtggcagccagca

tggaggtagatgtgcaggaaaagctagaacagctggagaccaacatggacaccttgtacactcgttttgcccgcctgctggccgagta

cacgggagcccagcagaagctcaagcagcgcatcacagttttggaaacgaagatgaagcagaataatgaggatgactccctgtcag

atgggatgaacagcccagagccacctgccgagaagccactcgagaaatccagaattacttctgaaggggagtatatccctctggatc

aaatagacatcaatgttgtcgacactagttctagaatggtgagcaagggcgaggagctgttcaccggggtggtgcccatcctggtcga

gctggacggcgacgtaaacggccacaagttcagcgtgtccggcgagggcgagggcgatgccacctacggcaagctgaccctgaa

gttcatctgcaccaccggcaagctgcccgtgccctggcccaccctcgtgaccaccttcggctacggcctgcagtgcttcgcccgctac

cccgaccacatgaagcagcacgacttcttcaagtccgccatgcccgaaggctacgtccaggagcgcaccatcttcttcaaggacgac

ggcaactacaagacccgcgccgaggtgaagttcgagggcgacaccctggtgaaccgcatcgagctgaagggcatcgacttcaagg

aggacggcaacatcctggggcacaagctggagtacaactacaacagccacaacgtctatatcatggccgacaagcagaagaacgg

catcaaggtgaacttcaagatccgccacaacatcgaggacggcagcgtgcagctcgccgaccactaccagcagaacacccccatcg

gcgacggccccgtgctgctgcccgacaaccactacctgagctaccagtccgccctgagcaaagaccccaacgagaagcgcgatca

catggtcctgctggagttcgtgaccgccgccgggatcactctcggcatggacgagctgtacaagtctagattttgctatgaaaatgaagt

t**taa**
