# Supplementary material for: Synthetic Light-Activated Ion Channels for Optogenetic Activation and Inhibition
Source: Front Neurosci. 2018 Oct 2;12:643. doi: 10.3389/fnins.2018.00643 (PMC6176052; doi:10.3389/fnins.2018.00643)
Supplement: Supplementary file 2 [file Data_Sheet_2.docx]

**Supplementary Videos**

**Video 1**. Single OLF-bP larva responds to 3 s blue light illumination (473 nm, 0.3 mW/mm^2^, indicated by a blue spot); play speed is 6 times of real speed.

**Video 2**. Single SthK-bP larva responds to 600 ms blue light illumination (473 nm, 0.3 mW/mm^2^, indicated by a blue spot); play speed is 6 times of real speed.

**Video 3**. Population of OLF-bP larvae respond to 1 s blue light illumination (470 nm, 1.6 mW/mm^2^, indicated by a white spot); play speed is 7 times of real speed.

**Video 4**. Population of SthK-bP larvae respond to 1 s blue light illumination (470 nm, 1.6 mW/mm^2^, indicated by a white spot); play speed is 14 times of real speed.
