# Supplementary material for: Synthetic Light-Activated Ion Channels for Optogenetic Activation and Inhibition
Source: Front Neurosci. 2018 Oct 2;12:643. doi: 10.3389/fnins.2018.00643 (PMC6176052; doi:10.3389/fnins.2018.00643)
Supplement: Supplementary file 3 [file Presentation_1.PPTX]

## Slide 1
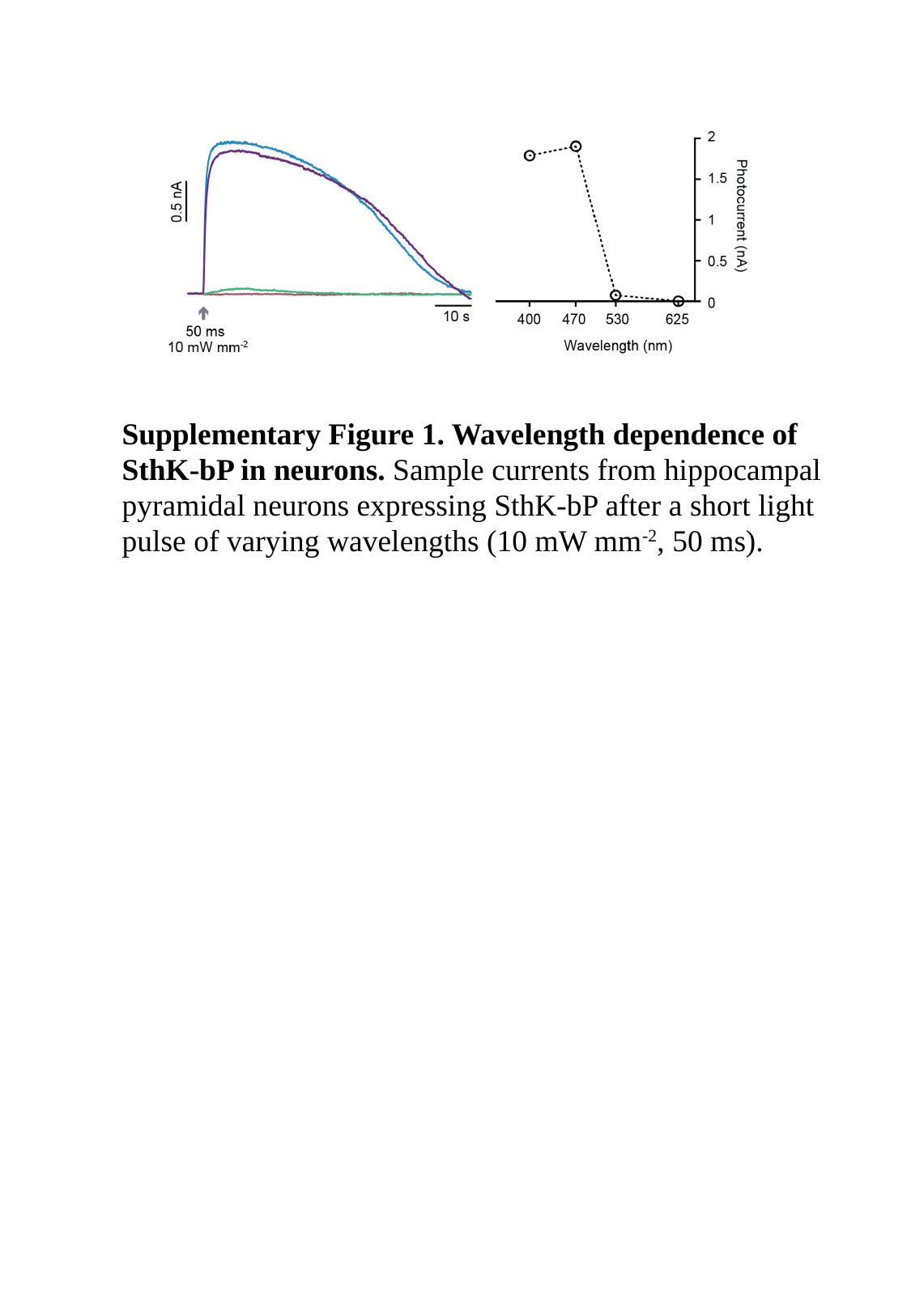

Supplementary Figure 1. Wavelength dependence of SthK-bP in neurons. Sample currents from hippocampal pyramidal neurons expressing SthK-bP after a short light pulse of varying wavelengths (10 mW mm-2, 50 ms).

## Slide 2
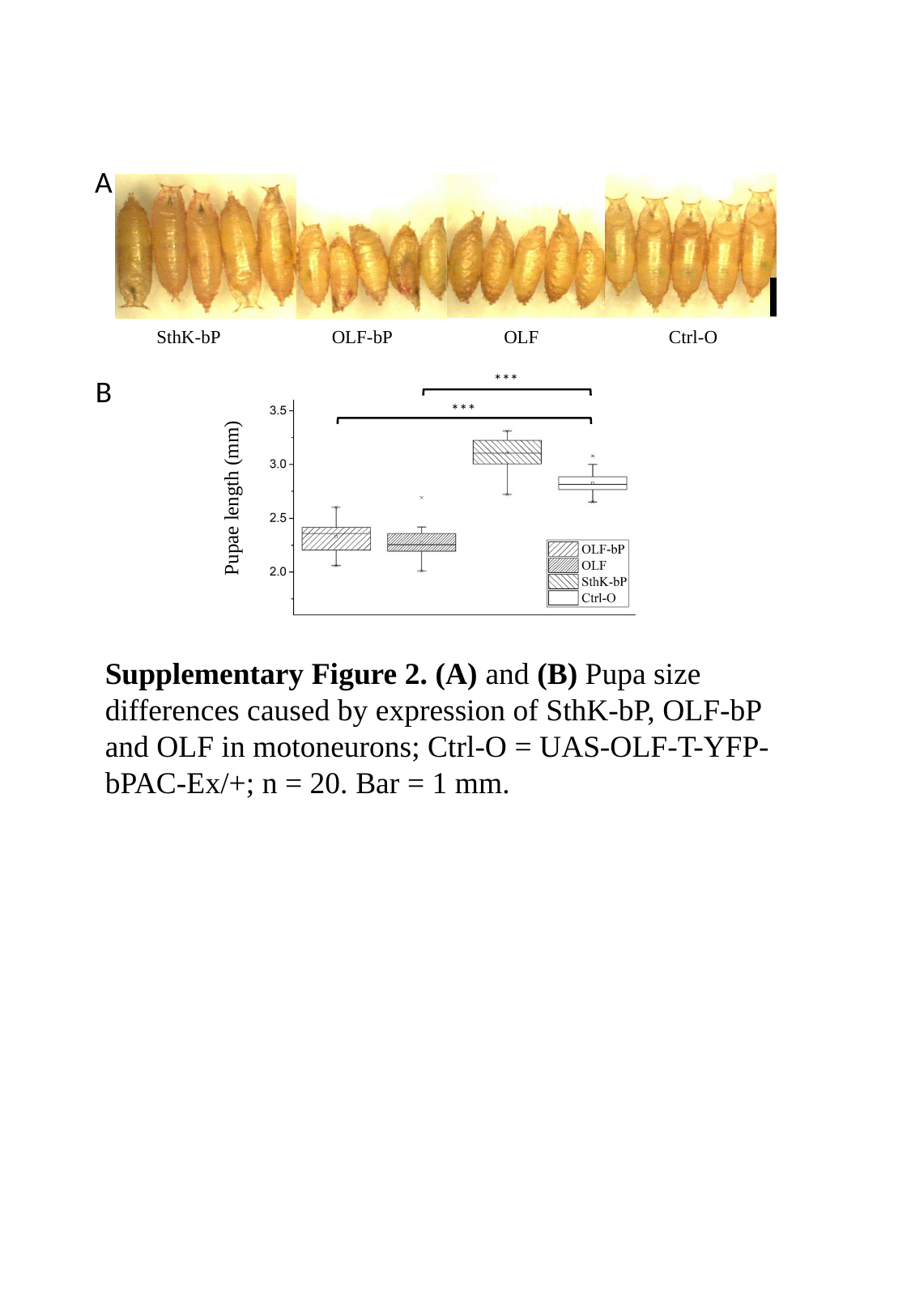

A
SthK-bP OLF-bP OLF Ctrl-O
***
***
Pupae length (mm)
B
Supplementary Figure 2. (A) and (B) Pupa size differences caused by expression of SthK-bP, OLF-bP and OLF in motoneurons; Ctrl-O = UAS-OLF-T-YFP-bPAC-Ex/+; n = 20. Bar = 1 mm.
